# Comparative Analysis of Mechanistic and Correlative Models for Global and Bhutan-Specific Suitability of Parthenium Weed and Vulnerability of Agriculture in Bhutan

**DOI:** 10.3390/plants14010083

**Published:** 2024-12-30

**Authors:** Sangay Dorji, Stephen Stewart, Asad Shabbir, Ali Bajwa, Ammar Aziz, Steve Adkins

**Affiliations:** 1 School of Agriculture and Food Sustainability, The University of Queensland, Gatton, QLD 4343, Australia; s.dorji@uq.edu.au (S.D.); a.abdulaziz@uq.edu.au (A.A.); 2 CSIRO Environment, Private Bag No. 5, GPO Box 1700, Hobart, TAS 7005, Australia; stephen.stewart@csiro.au; 3 Weeds Research Unit, Invasive Species Biosecurity, New South Wales Department of Primary Industries and Regional Development, Orange, NSW 2800, Australia; asad.shabbir@dpi.nsw.gov.au; 4 La Trobe Institute of Sustainable Agriculture and Food (LISAF), Department of Ecological, Plant and Animal Sciences, AgriBio, La Trobe University, Melbourne, VIC 3086, Australia; a.bajwa@latrobe.edu.au

**Keywords:** *Parthenium hysterophorus*, species distribution modelling, CLIMEX, random forest, climate change, invasive alien species, mechanistic models, correlative models

## Abstract

Parthenium weed (*Parthenium hysterophorus* L.) is one of the most noxious and fast-spreading invasive alien species, posing a major threat to ecosystems, agriculture, and public health worldwide. Mechanistic and correlative species distribution models are commonly employed to determine the potential habitat suitability of parthenium weed. However, a comparative analysis of these two approaches for parthenium weed is lacking, leaving a gap in understanding their relative effectiveness and ability to describe habitat suitability of parthenium weed. This study compared the mechanistic model CLIMEX with random forest (RF), the best-performing of a suite of correlative models. When compared against occurrence records and pseudo-absences, measured by area under the receiver operating characteristic curve, true skill statistic, sensitivity, and specificity, the results revealed higher performance of RF compared to CLIMEX. Globally, RF predicted 7 million km^2^ (2% of the total land mass) as suitable for parthenium weed, while CLIMEX predicted 20 million km^2^ (13%). Based on binary maps, RF and CLIMEX identified 67 and 20 countries as suitable, respectively. For Bhutan, globally trained RF predicted 8919 km^2^ (23% of the country’s total 38,394 km^2^) as currently suitable, with high suitability in the southern, west–central, central, and eastern districts, particularly along major highways. For the future, the 10 general circulation models downscaled to Bhutan showed a decrease in suitability across four scenarios (SSP126, SSP245, SSP370, SSP585) and three periods (2021–2050, 2051–2080, 2071–2100), with a northward shift in suitable habitats ranging from 2 to 76 km. Additionally, 2049 (23%) km^2^ of agricultural land is currently at risk of being invaded by parthenium weed. Correlative and mechanistic models are based on different niche concepts (i.e., realized and fundamental, respectively), and therefore combining them can provide a better understanding of actual and potential species distributions. Given the high suitability of parthenium weed under the current climate and its potential negative impacts in Bhutan, early action such as early detection and control of infested areas, regular survey and monitoring, and creating public awareness are proposed as risk mitigation strategies.

## 1. Introduction

Parthenium weed (*Parthenium hysterophorus* L.) is one of the most aggressive and rapidly spreading invasive alien species (IAS) known worldwide [1,2]. Originating from the Gulf of Mexico, including the southern United States of America and South America [3], parthenium weed is now reported from at least 92 countries [4], rapidly colonizing diverse forms of landscapes [5]. It poses severe threat to agriculture, with crop losses reported as high as 40% in rice and 90% in fodder [6,7]. It also causes damage indirectly by hosting pests and diseases [8,9]. The financial cost for its control and management is considerable, with India spending USD 3.66 billion annually [10]. Its high impact on livestock is also known as being reported in Australia, where parthenium weed reduced pasture availability and tainted milk and meat [11]. Parthenium weed is also a severe public health risk, causing dermatitis [12,13] and respiratory ailments like asthma, bronchitis, and rhinitis [14,15]. Additionally, it threatens native biodiversity by altering species composition through its strong allelopathy [16] and morphological and physiological characteristics [17], which allow it to outcompete and replace native plants [16,18].

Bhutan is one of the countries that are invaded by parthenium weed [19]. It is rapidly spreading and already detected in 19 of its 20 districts in the country [20]. The presence of this weed in Bhutan is a significant concern for the country’s rich biodiversity and small agriculture sector, as only about 7% of its total area is arable due to its mountainous terrain [21]. Climate change, which Bhutan is highly vulnerable to [22], further exposes the country to increased risk of biological invasions due to increased habitat suitability for certain IAS [23,24,25].

Species distribution models (SDMs) are widely used tools to study IAS ranges, assess potential habitat suitability, and manage biological invasions [26,27,28,29,30]. SDMs can be broadly categorized into two types: (i) correlative models and (ii) mechanistic models [31]. Correlative models work by finding relationships between species occurrences and environmental conditions, such as temperature and precipitation, and use the patterns identified in the data to predict where species will occur across landscapes. For example, if a species is found in regions with temperatures between 20 and 30 °C and precipitation between 500 and 1500 mm, the correlative models will identify similar areas as suitable habitats. Correlative models are further categorized as regression-based models (e.g., generalized linear models, generalized additive models), classification-based models (e.g., classification and regression tree), and complex models (boosted regression trees, random forests, MaxEnt) [32].

Correlative models are widely used in SDMs due to the availability of species records from citizen science and collaborative biodiversity databases (e.g., the Global Biodiversity Information Facility, GBIF) and widespread availability of global bioclimatic datasets (e.g., CHELSA [33], WorldClim [34]) since their introduction [35], as well as the ongoing development of user-friendly software and modelling tools that have greatly improved accessibility and ease of implementation. Further, random forest (RF) and MaxEnt are the two most widely used correlative models in SDMs because of their high predictive performance [36]. Ensemble modelling is also a common practice undertaken in correlative models to overcome the limitations of individual models [37].

The other category, mechanistic models, work by focusing on biological processes and factors that limit species’ growth and survival, such as temperature tolerance, water requirements, or frost tolerance. These models use cause-and-effect relationships to predict where a species can survive and thrive, and do not require species presence or occurrence records, as is the case for correlative models. While mechanistic models typically require detailed species-specific knowledge and can be complex, they provide valuable insights into the potential distribution of species based on fundamental biological processes [38], making them highly relevant for well-studied species like parthenium weed. CLIMEX is a popular software based on mechanistic approaches and is commonly applied in agricultural contexts [39,40,41]. It has also been used for predicting habitat suitability of parthenium weed [42,43]. Hybrid models that combine correlative and mechanistic models is an emerging practice [31,38,44,45].

Both correlative and mechanistic models have been independently implemented for parthenium weed; however, no study has been undertaken to compare their predictions. The global prediction of habitat suitability using correlative models determined vast stretches of suitable habitats for parthenium weed in multiple continents [46]. Similarly, the mechanistic model, CLIMEX, has been used to study suitable habitat for parthenium weed globally [42], in Africa [42,43], and in Asia [47]. These implementations were undertaken independently, and there is a gap in understanding their relative effectiveness, accuracy, and ability to describe invasion dynamics.

In Bhutan, parthenium weed modelling using correlative models has been inconsistent, predicting both increases [20] and decreases [48] in habitat suitability. These studies relied on WorldClim [34], a dataset that is widely used for SDMs but with limited accuracy in Bhutan when compared with locally calibrated models [49]. High-resolution (250 m) gridded historical climatologies (1986–2015) have been made available for Bhutan using national weather station observations [50]. This dataset more accurately reflected Bhutan’s complex topography and revealed considerable differences in temperature and precipitation compared to WorldClim [34] (e.g., inverted precipitation gradient with elevation). Utilizing this dataset, 10 general circulation models (GCMs) that have participated in the Coupled Model Inter-comparison Project Phase 6 (CMIP6) [51] were downscaled to develop future climate projections for Bhutan. With the availability of fine-resolution historical and future climate data for Bhutan, understanding the habitat suitability and impacts of climate change on parthenium weed can be greatly improved. Additionally, the impact of the predicted suitability of parthenium weed on agricultural land in Bhutan has not been assessed, and therefore, the implication of parthenium weed on the agriculture sector is not yet known.

The aim of this study was to investigate global habitat suitability for parthenium weed predicted by correlative and mechanistic SDMs, and the potential implications for agriculture in Bhutan under climate change. To address these aims, this study (1) modelled and compared the global habitat suitability of parthenium weed using CLIMEX and correlative models, (2) predicted the habitat suitability of parthenium weed and tracked the direction of spread in the future under climate change in Bhutan, and (3) identified agricultural areas predicted to be suitable for parthenium weed under current and future climate change scenarios in Bhutan.

## 2. Materials and Methods

### 2.1. Correlative Models

#### 2.1.1. Occurrence Data, Cleaning, and Spatial Thinning

Parthenium occurrence (longitude and latitude) was compiled from various sources (Supplementary Table S1). In total, 33,464 occurrences were obtained, but the Global Biodiversity Information Facility (GBIF) was the primary source, providing 27,968 occurrence records distributed at least in 30 native and 40 invaded countries (Supplementary Figure S2).

As publicly available data are prone to errors, GBIF data were thoroughly checked for locations without coordinates, duplicate coordinates, incomplete coordinates, imprecise coordinates, unlikely coordinates, and records with zero abundance or status absent but coordinates recorded using *dismo* [52] and *scrubr* [53] packages in R version 4.4.0 [54]. To reduce spatial autocorrelation and sampling bias [55], occurrence records were geographically and environmentally thinned using the *flexsdm* [56] package in R version 4.4.0 [54], which returned 7935 occurrence records, which were then used for modelling.

#### 2.1.2. Bias Layers and Generation of Pseudo-Absences

**Figure 1:**
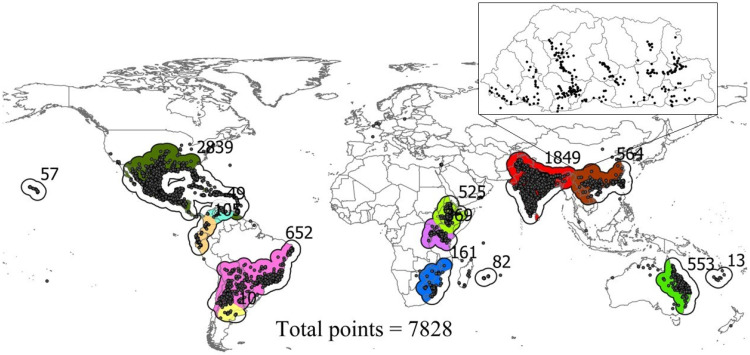
Pseudo-absences were randomly generated within each of the 14 bias layers (colored regions), equal to the occurrences enclosed by each bias layer, indicated by the number beside each bias layer. Solid lines around each bias layer represent the buffers dissolved, which were used to crop one of the “WorldClim” bioclimatic layers to generate bias layers. The inset map shows the current distribution of parthenium weed in Bhutan.

Since SDMs based on occurrences and pseudo-absences tend to perform better than presence-only SDMs [57], this study implemented this approach. As the data collected from various sources (Supplementary Table S1) contained presence-only data, pseudo-absences were generated as follows. Regional clusters of occurrences were first identified. Circular buffers with a minimum radius of 10 km and a maximum of 400 km were then generated around the occurrences using the *terra* package in R version 4.4.0 [54]. Buffers were then dissolved, and one of the WorldClim [34] bioclimatic variables with 1 km resolution was cropped, which were then used as layers from which pseudo-absences were generated (colored regions, Figure 1). The term “bias layers” is used to indicate these regions in this study. Using these bias layers, occurrences were intersected, and counts were obtained for each bias layer. Based on the counts captured by each bias layer, pseudo-absences were generated randomly within each bias layer using the *terra* package in R version 4.4.0 [54]. The number of pseudo-absences generated for each bias layer is given in the Figure 1, in which the number beside each bias layer corresponds to the number of pseudo-absences generated. In total, 7828 points were randomly generated as pseudo-absences (Figure 1).

#### 2.1.3. Modelling, Model Evaluation, and Predictions

The dataset for modelling was built as follows. A total of 19 bioclimatic variables were obtained from WorldClim [34], https://www.worldclim.org/ (accessed on 3 August 2023). They were tested for multicollinearity using the Spearman method with the threshold set at 0.7, which resulted in the selection of six bioclimatic variables (Supplementary Table S2). The 19 bioclimatic variables were also reviewed for their use in parthenium weed SDM and relevance ecologically [20,23,46,48,58] (Supplementary Table S3). These processes resulted in the selection of 10 bioclimatic variables (Table 1) for modelling. From these 10 bioclimatic variables, values were extracted at each of the occurrence and pseudo-absence locations to build a dataset for modelling.

For training, the dataset was split using repeated *k*-fold cross-validation, which is a rigorous method that divides the dataset into *k* equally sized subsets called “folds”, in which the model is tested using one fold and trained with the remaining folds (*k* − 1), and this is repeated until all the folds have been used for testing [59,60]. This method was implemented using the *flexsdm* package in R version 4.4.0 [54] with five folds (*k* = 5) and replicated five times, resulting in a total of 25 models.

Numerous evaluation metrics are available for assessing model performance in SDMs [32], each with strengths and limitations. In this study, we implemented four widely used evaluation metrics: area under the receiver operating characteristic curve (AUC), true skill statistic (TSS), sensitivity (true positive rate), and specificity (true negative rate), incorporating both threshold-dependent and threshold-independent metrics to ensure a comprehensive evaluation. The AUC, which evaluates model performance across all possible classification thresholds, eliminates the need for selecting a single, arbitrary threshold [61], and is therefore considered a threshold-independent evaluation metric [62]. Based on the AUC, models are classified as failed (0.5–0.6), poor (0.6–0.7), fair (0.7–0.8), good (0.8–0.9), and excellent (0.9–1) [62]. In contrast, the TSS is a threshold-dependent metric that measures the ability of a model to correctly distinguish between presences and absences. Its value ranges from −1 to +1, where +1 indicates perfect agreement and −1 perfect disagreement [63]. Sensitivity represents the proportion of correctly predicted presences, while specificity represents the proportion of correctly predicted absences, and they are independent of each other and not affected by prevalence [23,62].

To predict habitat suitability, we used the generalized additive model (GAM), generalized boosted model (GBM), and RF, which are widely used in SDMs [57,64]. The best model was selected to predict habitat suitability. For the global model, habitat suitability was predicted only for the current climate, while for Bhutan, it was predicted both for the current and future climates. The future predictions for Bhutan included 10 GCMs, four scenarios (SSP126, SSP245, SSP370, SSP585), and three periods (2021–2050, 2051–2080, 2071–2100) developed for Bhutan [65]. Additionally, agricultural areas predicted to be suitable for parthenium weed were determined under the current and future climates by overlaying the agricultural land polygons [21] with the suitability maps and extracting the overlapping areas.

We evaluated the agreement and uncertainty of the 10 GCMs model predictions using two metrics: TAI, which measures the model agreement, and TSD, which is an indicator of model agreement and uncertainty [66]. The TAI values range from −1 (unsuitable, where all models agree) to 1 (suitable, where all models agree). These metrics were applied only to Bhutan.

#### 2.1.4. Suitability Tracking of Parthenium Weed in Bhutan Under Future Climate Change

The direction and habitat suitability of parthenium weed for 10 GCMs under four scenarios (SSP126, SSP245, SSP370, SSP585) and three periods (2021–2050, 2051–2080, 2071–2100) were determined using the standard deviation ellipse (SDE). The SDE provides a summary of the spatial characteristics of data points, including the central tendency, dispersion, and directional trends [67]. The center of the ellipse represents the mean center of the data points. The dispersion of the data is indicated by the axes of the ellipse, which depict the standard deviation of the points in the north–south and east–west directions. The length of each axis indicates the degree of spread in that direction, with longer axes suggesting greater dispersion. The directional trend is indicated by the orientation of the tilt. The binary maps were converted into values and coordinates of suitability values equal to or above the threshold value were used for building the SDE. The ellipses were created at one standard deviation using the *aspace* [68] package in R version 4.4.0 [54].

### 2.2. Mechanistic Model—CLIMEX and Parameter Settings, Model Evaluation, and Suitability Prediction

For mechanistic modelling, the CLIMEX software (Climex and Dymex Suite 4.0.2) was used to determine the suitability of parthenium weed. The CLIMEX calculates the suitability of a species in a particular location by combining growth and stress indices [69,70] to give suitability scores, called the “Ecoclimatic Index (EI)”, ranging from the lowest, “0”, to the highest “100”, representing “not suitable” to “highly suitable”, respectively [42]. The settings (Supplementary Table S4) used in CLIMEX in this study were originally applied in the parthenium distribution modelling by Shabbir, et al. [47].

The performance of CLIMEX was evaluated using the same metrics as the correlative models above. The occurrence and pseudo-absence values were extracted from the suitability map under the current climate change and the metrics were calculated using the *mecofun* [71] package in R version 4.4.0 [54].

For suitability prediction, we utilized the CM10-75H-V1.2mm climate dataset at 10′ resolution centered on 1975 [72], which is integrated within the CLIMEX software. The suitability values, represented by the Ecoclimatic Index (EI), were exported as comma-separated values (“.csv”), along with the corresponding coordinates for subsequent mapping and analysis in ArcGIS Pro.

### 2.3. Suitability Comparison Between RF and CLIMEX

To compare the global suitability predictions between RF and CLIMEX, continuous maps were converted into binary maps using the threshold that maximized sensitivity and specificity. Using the World Bank’s official administrative boundaries obtained from https://datacatalog.worldbank.org/ (accessed on 20 October 2024), suitable areas with the names of the countries were extracted and compared with each other.

### 2.4. Field Validation of RF Performance

The performance of RF was validated in the field by visiting Bhutan in April 2024. Areas predicted as suitable were visited and observed for parthenium weed’s presence. Given the time and resource constraints, the validation focused exclusively on areas predicated as suitable habitats, with particular emphasis on locations along national highways, where high suitability was predicted.

### 2.5. Use of Artificial Intelligence

During the preparation of this article, OpenAI’s ChatGPT, an AI-based language model, was used to increase the clarity and organization of certain sentences and paragraphs. In particular, it was used to check grammar, sentence fluency, and clear and concise explanation of complex ideas. Its application facilitated the polishing of written content, contributing to the overall readability and coherence of the manuscript. However, all outputs and interpretations were reviewed and edited by the authors to ensure accuracy and appropriateness prior to finalization.

## 3. Results

### 3.1. Model Evaluation and Selection

The threshold-independent evaluation metric (AUC) ranged from 0.81 in GAMs and to 0.92 in RF, while the threshold-dependent metric (TSS) ranged from 0.49 in GAMs to 0.68 in RF (Table 2). The sensitivity (true positive rate) ranged from 0.78 in generalized boosted models (GBMs) to 0.86 in RF, while the specificity (true negative score) ranged from 0.69 in GAMs to 0.82 in RF. In comparison, CLIMEX gave an AUC score of 0.69, with sensitivity and specificity at 0.61 and 0.70, respectively, and a TSS score of 0.30 (Table 2). Overall, RF was the best-performing model and rated as “excellent” [62]. Consequently, it was selected to predict habitat suitability for parthenium weed at both the global and Bhutan levels and was compared against CLIMEX, the only mechanistic model used in the analysis.

### 3.2. Variable Importance and the Effect of Predictor Variables

**Figure 2:**
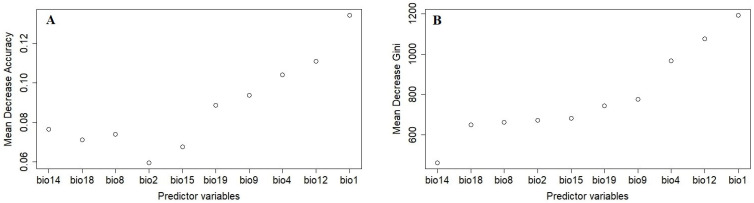
A variable importance plot of predictor variables based on mean decrease accuracy (**A**) and mean decrease Gini (**B**). The mean decrease accuracy plot shows how much the model’s predictive accuracy drops when a variable is excluded, with higher values showing greater importance. The mean decrease in Gini measures how much each variable contributes to the node purity within the decision trees, where higher values indicate a stronger role in improving classification performance. Abbreviation: bio1 = annual mean temperature (°C), bio2 = mean diurnal range (°C), bio4 = temperature seasonality (°C), bio8 = mean temperature of the wettest quarter (°C), bio9 = mean temperature of the driest quarter (°C), bio12 = annual precipitation (mm), bio14 = precipitation of the driest month (mm), bio15 = precipitation seasonality (coefficient of variation) (dimensionless), bio18 = precipitation of the warmest quarter (mm), bio19 = precipitation of the coldest quarter (mm) [35].

The importance of predictor variables was measured based on the mean decrease in accuracy (Figure 2A) and the mean decrease in Gini (Figure 2B). The mean decrease in accuracy plot shows how much the model’s predictive accuracy drops when a variable is excluded, with higher values showing greater importance, while the mean decrease in Gini measures how much each variable contributes to the node purity within the decision trees, where higher values indicate a stronger role in improving classification performance. Among the 10 predictor variables used in parthenium weed habitat suitability modelling, annual mean temperature (bio1) was the most important, followed by annual precipitation (bio12), temperature seasonality (bio4), and so on (Figure 2).

### 3.3. Predicting Habitat Suitability of Parthenium Weed by Random Forest and CLIMEX

#### 3.3.1. Correlative Model, Random Forest Prediction

**Figure 3:**
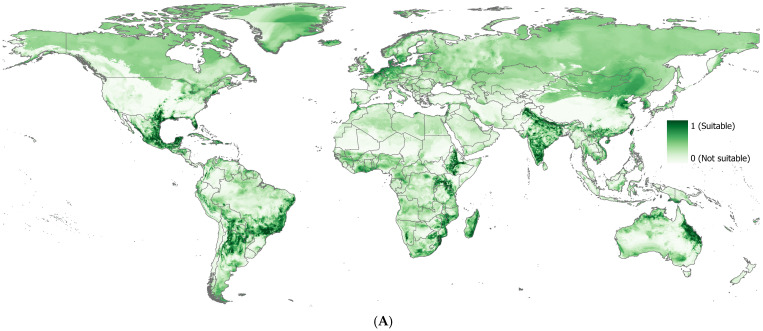

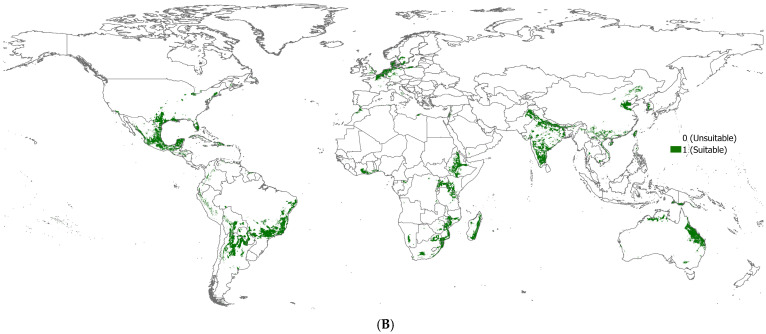
(**A**) The global suitability of parthenium weed predicted by random forest, where darker shades of green indicate higher suitability; and (**B**) the binary suitability map. The binary classification was generated using the threshold value 0.588, which maximized sensitivity (true positive rate) and specificity (true negative rate).

The correlative model, RF, produced spatially complex prediction patterns for the global distribution of parthenium weed; however, certain high-latitude (e.g., Greenland and Scandinavia) and temperate regions (e.g., Canada and the northern United States) showing poor predictive accuracy (Figure 3A,B). The total global suitability area for parthenium weed is ca. 7 million km^2^ (2%). Asia has the largest total area at ca. 2 million km^2^, with India (0.9 million km^2^) and China (0.4 million km^2^) contributing the most. South America is dominated by Brazil (0.8 million km^2^) and Argentina (0.5 million km^2^), making it the second-largest region. Africa has Ethiopia (0.3 million km^2^) as the country with the largest suitability, followed by Mozambique (0.2 million km^2^). Oceania is led by Australia (0.6 million km^2^), which accounts for the majority of the region’s land area. Europe has Germany (0.1 million km^2^) and France (0.04 million km^2^) as the leading contributors to Europe’s total area of 0.3 million km^2^ suitable to parthenium weed.

#### 3.3.2. Mechanistic Model, CLIMEX Prediction

**Figure 4:**
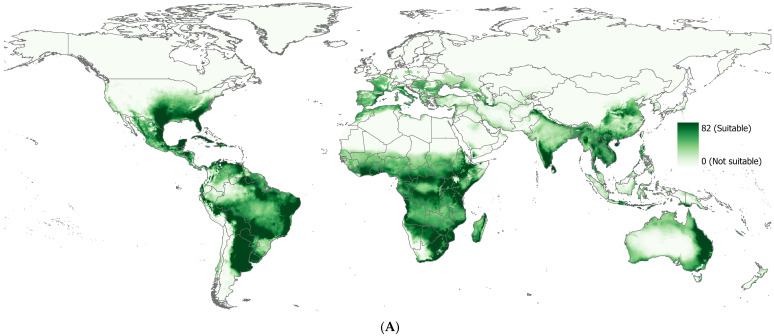

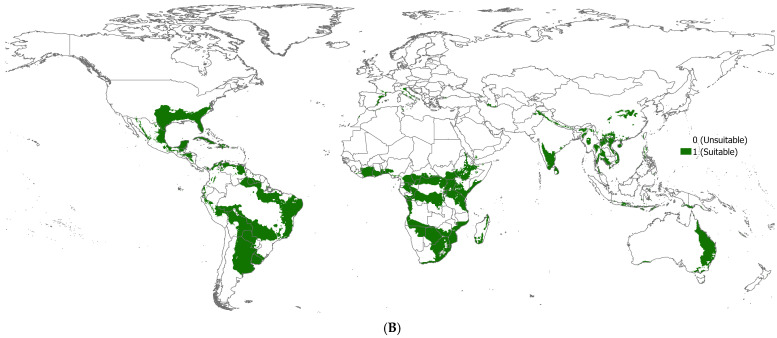
(**A**) The global suitability of parthenium weed predicted by the CLIMEX model, where darker shades of green indicate higher suitability; and (**B**) the binary suitability map. The binary classification was generated using the threshold value of 26, in which the sum of specificity and sensitivity was maximized.

The CLIMEX model predicted areas with high suitability predominantly in tropical and subtropical zones (Figure 4A,B). The global suitability of land area across regions and countries to parthenium weed is ca. 20 million km^2^ (13%). Africa accounts for the largest suitability, with a total area of ca. 7 million km^2^. Significant contributors include the Democratic Republic of the Congo (1 million km^2^), South Africa (0.7 million km^2^), Tanzania (0.5 million km^2^), and Ethiopia (0.5 million km^2^). Asia contributes a total area of ca. 2 million km^2^. India dominates the region, with 0.5 million km^2^, followed by the People’s Republic of China (0.4 million km^2^) and Thailand (0.2 million km^2^). Europe has a total land area of approximately 0.13 million km^2^. Major contributors include Italy (0.06 million km^2^), Spain (0.05 million km^2^), and France (0.02 million km^2^).

#### 3.3.3. Suitability Comparison Between Random Forest and CLIMEX

**Figure 5:**
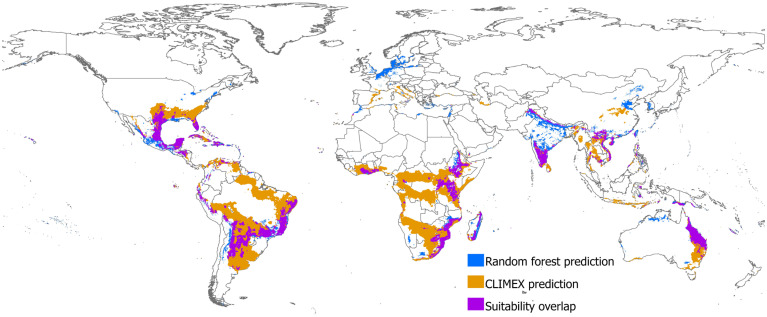
Comparison of predicted suitability of parthenium weed between random forest and CLIMEX. The binary map of random forest (blue) is overlaid on the binary map of CLIMEX (orange) with their suitability overlap (violet). The binary maps were generated using a threshold value that maximized the sensitivity and specificity, which was 0.588 for RF and 26 for CLIMEX.

The binary maps of RF were overlaid on CLIMEX (Figure 5), highlighting differences and overlaps. The binary maps were generated using the threshold value that maximized sensitivity and specificity, which was 0.588 and 26 for RF and CLIMEX, respectively. Based on these respective thresholds, RF identified 165 countries globally as suitable for parthenium weed, while CLIMEX predicted suitability in 118 countries. The overlap area between the CLIMEX and RF is 5 million km^2^, which ca. 25% of the CLIMEX area and 71.4% of the RF area. Despite predicting fewer countries, CLIMEX predicted 13 million km^2^ more suitable area compared to RF. Notably, RF identified 67 countries as suitable that were not predicted by CLIMEX (Supplementary Table S5). These included countries mainly in Africa (Burkina Faso, Chad, Libya, Mali, Senegal), Asia (Bhutan, Cyprus, Hong Kong, Iraq, Israel, Japan, Jordan, Lebanon, Macau, Malaysia, North Korea, Oman, Palestine, Singapore, South Korea, Syria, United Arab Emirates), and Europe (Austria, Belgium, Czech Republic, Denmark, Estonia, Finland, Germany, Gibraltar, Greece, Latvia, Lithuania, Malta, Norway, Poland, Sweden, Switzerland, Ukraine, United Kingdom) (Supplementary Table S5). Conversely, CLIMEX predicted 20 countries as suitable that were not identified by RF (Supplementary Table S5). These included countries in Africa (Central African Republic, Djibouti, Equatorial Guinea, Gabon, Liberia, Sierra Leone, Sudan, São Tomé and Príncipe), Asia (Afghanistan, Azerbaijan, Georgia), Europe (Albania, Bosnia and Herzegovina, Croatia, Monaco, Montenegro, Vatican City), North America (Guantanamo Bay Naval Base, Panama), and South America (Uruguay) (Supplementary Table S5).

### 3.4. Predicted Suitability and Changes of Parthenium Weed in Bhutan Under Current and Future Climate Change and Uncertainty

Under current climatic conditions, 23% (8919 km^2^) of Bhutan was predicted as suitable (Table 3). In the future, there is considerable variation in suitability across models, scenarios, and periods, but it typically declined from the 2021–2050, through the 2051–2080, to the 2071–2100 period compared to the current climate (Table 3, Supplementary Table S6). In the 2021–2050 period, the suitable area predicted by 10 GCMs that have participated in CMIP6 ranged from 18% (1579 km^2^), as predicted by CNRM-CM6-1 under the SSP585 scenario, to 60% (5366 km^2^), as predicted by NorESM2-MM, as well as under SSP585 (Supplementary Table S6). For the 2051–2080 period, MPI-ESM1-2-LR under the SSP585 scenario predicted the lowest area of suitability (6%; 557 km^2^), while ACCESS-CM2 predicted the highest suitability under SSP126 (56%; 5020 km^2^) (Table 3, Supplementary Table S6). In the 2071–2100 period, the least amount of suitable area was predicted by MPI-ESM1-2-LR under SSP585 (2%; 143 km^2^), while MIROC6 predicted the highest suitability under SSP126 (61%; 5408 km^2^) (Supplementary Table S6).

**Figure 6:**
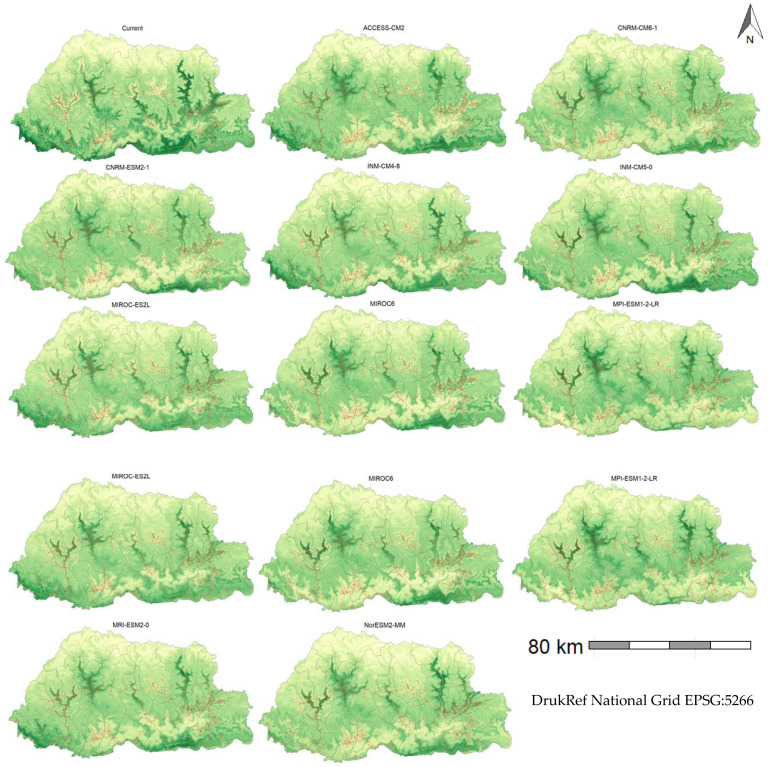
The spatial map of predicted parthenium weed suitability in Bhutan under the current (1985–2015) and future (2051–2080) climate based on the SSP245 scenario, where darker shades of green indicate higher suitability. Higher suitability is typically found along the national highways (red lines), particularly in Punakha to Wangduephodrang, Trongsa to Zhemgang, Mongar to Lhuentse, Mongar to Trashigang, and Trashigang to Trashiyangtse. The names above each plot indicate the names of the general circulation models. The red lines are road networks.

To further elucidate the results and give more perspective, suitability prediction is presented for the 2051–2080 period (Table 3) and maps for the same period but under the SSP245 scenario only (Figure 6). This scenario and period were chosen because Bhutan is predicted to exceed the Paris Agreement of limiting global warming to 1.5 °C [73] within this timeframe, making it particularly relevant for Bhutan’s climate analysis. Additionally, the SSP245 scenario, representing the “middle of the road” with medium challenges to mitigation and adaptation [74], is appropriate for Bhutan. It aligns with Bhutan’s development philosophy of gross national happiness, balancing economic growth with preservation of culture and environment [75].

In the 2051–2080 period (Table 3), among the GCMs, ACCESS-CM2 showed considerable values across all pathways, with SSP126 (5019 km^2^) and SSP585 (3661 km^2^) being the highest. CNRM-CM6-1 and CNRM-ESM2-1 exhibited relatively low values for SSP370 and SSP585 compared to other GCMs. INM-CM4-8 performed consistently high across all pathways, particularly SSP245 (3347 km^2^) and SSP370 (3039 km^2^). MIROC6 and MRI-ESM2-0 showed substantial values in SSP126, with 3898 and 4224 km^2^, respectively, and maintained higher variability across other pathways. NorESM2-MM stood out for SSP370, with a peak value of 4866 km^2^, while other scenarios displayed lower projections. Overall, the data indicated substantial variability in GCM performance under different SSPs, highlighting contrasting climate responses depending on the pathway scenario.

The predictive maps of current suitability of parthenium weed under the current climate (1986–2015) showed high suitability along the national highways (Figure 6, Supplementary Figures S2 and S3). These areas were primarily located along national highways, including routes from Punakha to Wangduephodrang, Trongsa to Zhemgang, Mongar to Lhuentse, Mongar to Trashigang, and Trashigang to Trashiyangtse, as well as along the southern borders of Chukha, Pema Gatshel, Samtse, and Zhemgang districts (Supplementary Figures S2 and S3). In the future, high suitability was still predicted along these same highways, albeit with reduced intensity (Figure 6, Supplementary Figures S2 and S3).

**Figure 7:**
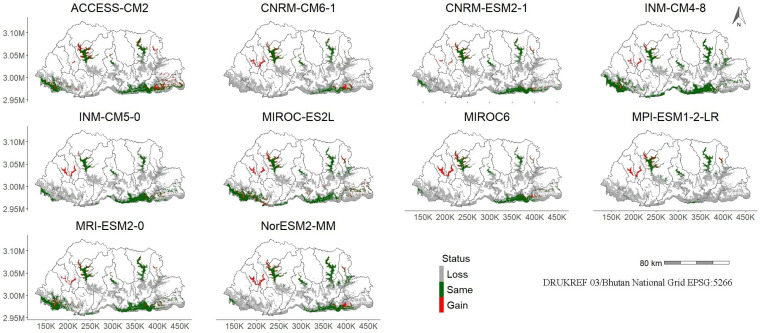
Projected changes in parthenium weed distribution under the SSP245 climate scenario for the 2051–2080 period. Gray regions represent areas where the predicted distribution is expected to decrease, while green and red regions highlight areas of no change and gain, respectively. Notably, areas of increased suitability were predicted along highways in Paro and Thimphu. Note: “K” and “M” in the *x*- and *y*-axis denote a thousand (×1000) and a million (×1,000,000) meters, respectively.

Changes in the predicted future suitability compared to the current distribution showed areas of increased suitability along the highways in Paro and Thimphu and some areas in the Punakha, east, and southeastern districts (Figure 7, Supplementary Figures S2 and S4). Generally, there is a higher loss of suitability under higher emission scenarios of SSP370 and SSP585 compared to the lower emission scenarios of SSP126 and SSP245 towards the middle and end of the century.

**Figure 8:**
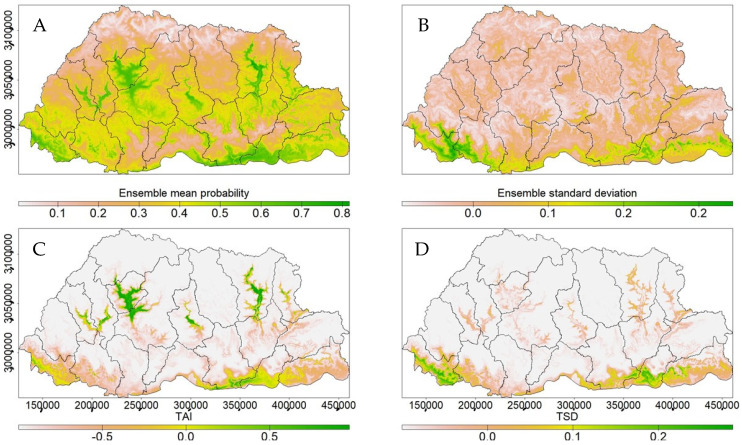
The congruence and uncertainty in predictions across 10 GCMs were assessed using the ensemble mean probability (**A**), ensemble standard deviation (**B**), Threshold Agreement Index (**C**), and threshold-scaled standard deviation (**D**). The darker green areas indicate strong agreement as well as uncertainty.

Model agreement and uncertainty across 10 GCMs indicated strong agreement, as measured by the ensemble mean probability (Figure 8A) and ensemble standard deviation (Figure 8B), respectively. All the models agreed on higher suitability, particularly in the southern, central, and eastern regions, with uncertainty in the southern and southeastern regions. Additionally, the Threshold Agreement Index (TAI) (Figure 8C)**,** which measured how the models agreed above or below the optimal classification threshold, indicated that more than 50% (values > 0.5) of the models agreed above this threshold. The threshold-scaled standard deviation (TSD) (Figure 8D) further measured variability around this threshold and highlighted the uncertainty in the southern regions.

### 3.5. Potential Impact of the Predicted Suitability on Agricultural Land in Bhutan Under Current and Future Climate Change

Under current climate conditions, model estimated that ca. 23% (2049 km^2^) of agricultural land will be potentially impacted by parthenium weed (Table 4). In future scenarios, the potential impact on agricultural land varied by model, scenario, and period, but overall, there was a decrease compared to the current suitability (Table 4, Supplementary Table S7). For the 2021–2050 period, the potential impact ranged from 30% (475 km^2^) in CNRM-CM6-4 under SSP585 to 30% (1608 km^2^) in NorESM2-MM under the same scenario (Supplementary Table S7). In the 2051–2080 period, it ranged from 38% (209 km^2^) in MPI-ESM1-2-LR under SSP585 to 28% (1420 km^2^) in ACCESS-CM2 under SSP126 (Supplementary Table S7). For the 2071–2100 period, the lowest potential impact was predicted by MPI-ESM1-2-LR at 20% (28 km^2^) under SSP585, while MIROC6 predicted the highest at 30% (1603 km^2^) under SSP126 (Supplementary Table S7). As an illustration, the potential impact on agricultural land under the four scenarios for 2051–2080 is given below (Table 4). In general, higher emission scenarios corresponded with lower potential impact on agricultural land.

**Figure 9:**
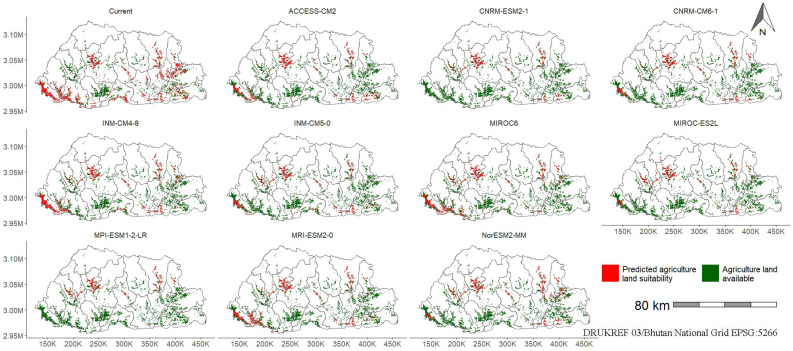
Maps of the potential impact of parthenium weed on agricultural land in Bhutan under the current and future climate of the SSP245 scenario and 2051–2080 period. The colored areas indicate the total agricultural land available in the country. Green indicates agricultural areas unlikely to be at risk, while red indicates areas likely to be at risk to parthenium weed. Note: “K” and “M” in the *x*- and *y*-axis denote a thousand (×1000) and a million (×1,000,000) meters, respectively.

The spatial analysis of agricultural land likely to be impacted by parthenium weed in Bhutan indicates that under the current climate, agricultural land in the southwest (Samtse and Dagana districts), central (Trongsa, Punakha, Wandguephodrang, and Zhemgang districts), and eastern districts is predicted to be at risk (Figure 9, Supplementary Figures S2 and S5). In the future, agricultural land in the west–central (Punakha and Wangduephodrang) and some areas in central (Zhemgang) and eastern districts—mainly Lhuentse and some portions of Samdrupjongkhar—is predicted to be at risk (Figure 9, Supplementary Figures S2 and S5). Importantly, core agricultural land in Paro, Thimphu, and west–central Bhutan (Punakha and Wangduephodrang) is predicted to be at risk to parthenium weed (Figure 9, Supplementary Figures S2 and S5). As an example, the potential impact on agricultural land by parthenium weed is illustrated below (Figure 9) for the SSP245 scenario under the 2051–2080 period.

### 3.6. Parthenium Weed Distribution Direction Under Climate Change

**Figure 10:**
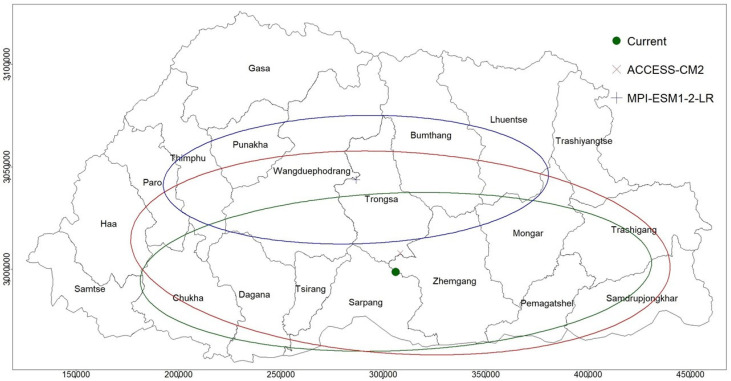
The standard deviational ellipse (SDE) and the mean location of parthenium weed distribution. Under the current climate (green), SDE is oriented towards the northeast direction at an angle of 87° (angle of rotation). In the future under the SSP245 scenario and during the 2051–2080 period, the two models that showed the lowest and the highest shift in the mean locations, ACCESS-CM2 (red) and MPI-ESM1-2-LR (blue), predicted a shift of 7 and 50 km northward, respectively.

Under the current climate scenario, the standard deviational ellipse (SDE) for parthenium weed distribution is oriented towards the northeast, with a clockwise rotation angle of 87° (Figure 10, green ellipse). In future projections, all models show a northward shift in the mean location of parthenium weed distribution (Figure 10, Supplementary Figure S6). During the 2021–2050 period, the shift in mean location ranges from a minimum of 2 km in INM-CM5-0 under SSP126 to a maximum of 39 km in NorESM2-MM under SSP370 (Supplementary Table S8). For the 2051–2080 period, the shift extends from 4 km (CNRM-ESM2-1) under SSP126 to 65 km (MPI-ESM1-2-LR) under SSP370. Similarly, in the 2071-2100 period, the shift ranges from 2 km (INM-CM5-0) under SSP126 to 76 km (MPI-ESM1-2-LR) under SSP370 (Supplementary Table S8).

For illustration and clarity, the SDE for the future is given below for the two models, ACCESS-CM2 (red) and MPI-ESM1-2-LR (blue), which predicted the lowest and the highest shifts in the mean location under the SSP245 scenario, the 2051–2080 period (Figure 10). The distribution is generally oriented from the southwest to northeast and the southeast to northwest directions. The lowest shift in the mean location of parthenium weed distribution is about 7 km in ACCESS-CM2 to the largest shift of about 50 km in MPI-ESM1-2-LR towards the north.

### 3.7. Field Validation of RF Prediction

**Figure 11:**
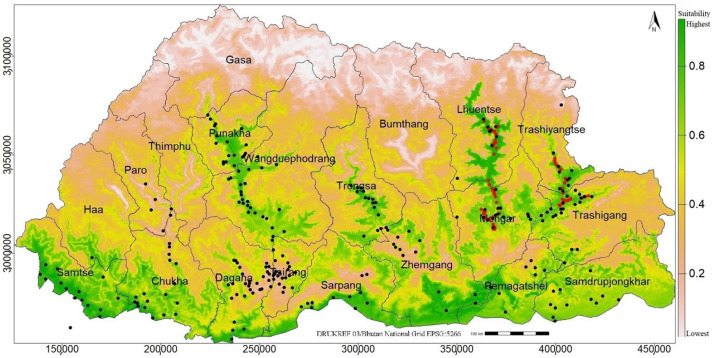
The map of Bhutan showing the predicted suitability of parthenium weed under the current climate (higher green intensity indicating higher suitability), which was validated through field visits. The red points (n = 92) represent locations where parthenium weed was detected during field visits conducted in April 2024. The black dots indicate parthenium weed occurrence records used for model calibration, sourced from multiple datasets (Supplementary Table S1).

There was strong agreement between the model predictions and field verifications for all the fields visited (n = 92), as indicated by the red points (Figure 11). Due to time and resource constraints, only the eastern part of Bhutan was visited. The strong alignment between field-verified occurrences and the model predictions highlighted the reliability of the RF model for predicting parthenium weed habitat in Bhutan.

## 4. Discussion

### 4.1. Occurrences, Model Evaluation, and Selection

The global habitat suitability of parthenium weed was most accurately predicted using RF compared to occurrence records and pseudo-absences, which have also been effective in many previous SDM studies [76,77,78,79]. RF was therefore chosen for predicting the global and Bhutan-specific distribution of parthenium weed, rather than the alternative correlative models, or the ensemble of algorithms used in the study (Table 2). Since one of the main factors that affect the model performance is related to background data [80], several strategies were undertaken to select it. To deal with the well-known phenomenon of artificial inflation of AUC due to the broad background extent with limited real-world relevance [46,81], RF was trained with a narrower background extent (Figure 1), as used in previous studies [46]. CLIMEX had the lowest performance when compared against occurrence and pseudo-absence records (Table 2) reported by others [82]. Although this approach of evaluating mechanistic models like CLIMEX is not a common practice, it was implemented in this study to facilitate direct comparison, and such evaluation is also recommended [82], as visual assessments are often subjective [83,84]. However, this does not necessarily mean CLIMEX was inferior to RF because metrics alone may not capture the ecological realism of predictions. Additionally, these two models operate differently. CLIMEX is based on a species’ fundamental niche, encompassing the full range of environmental conditions under which a species can survive and reproduce [85]. In contrast, the realized niche is a subset of the fundamental niche and accounts for interactions with other species, dispersal limitations, and historical factors that influence where a species is found [86].

### 4.2. Global Habitat Suitability of Parthenium Weed Under the Current Climate

The global habitat suitability of parthenium weed predicted using the 10 bioclimatic variables at a 2.5 min resolution (ca. 4.5 km at the equator) were carefully selected not only based on their low collinearity (Supplementary Table S2) but also on their ecological relevance and literature review (Supplementary Table S3). This multistep selection of environmental predictors was undertaken to ensure that the ecologically meaningful variables were not discarded by the machine. Among the 10 variables, annual mean temperature (bio1) was the most important for determining global parthenium weed habitat suitability (Figure 2), in line with other studies [23,25].

Overall, there was a general agreement between predicted habitat suitability of RF and actual observations. However, poor predictive accuracy was observed in certain regions, mainly in high-latitude areas, including Greenland and Scandinavia, and temperate regions, including Canada, parts of Northern and Western Europe, and the northern United States, similar to others [46]. The suitability maps produced by RF (Figure 3) and CLIMEX (Figure 4) showed differences in the predicted suitability areas (Figure 5). RF projected smaller and more fragmented suitability areas but identified 67 additional countries as suitable compared to CLIMEX, primarily in the temperate regions of Western Europe. While the temperatures may be too cold for parthenium weed, the occurrence of parthenium weed populations has been reported in Germany [87], Romania [88], Poland [89,90], Belgium [91,92,93], Sweden [94,95], France [96], and the Netherlands [97], but it is not clear whether these populations have established and persisted or perished. Seed germination at extreme winter temperature of 2.6 °C was also reported [98]. Conversely, CLIMEX predicted 20 countries as suitable that were not predicted by RF. This difference in suitability was attributed to the threshold used to produce binary maps. RF classified all countries below the threshold value of 0.588 as unsuitable, while CLIMEX used an ecoclimatic index of 26 for the same. However, an ecoclimatic index between 21 and 50 was classified as highly suitable by Kriticos, et al. [42]. The difference was also due to how these two models operate. RF is based on the realized niche, which is a subset of the fundamental niche and includes interactions with other species, dispersal limitations, and historical influences that determine the species’ actual distribution [86]. Correlative models, dependent on occurrence data, reflect this realized niche. As a result, they often predict narrower distributions, constrained by observed environmental associations rather than the full physiological tolerance range of the species [99]. CLIMEX works based on a species’ fundamental niche and aims to represent this by using physiological data to predict broader potential areas where a species could theoretically exist, even if it has not been observed there yet [85]. Nevertheless, habitat suitability predicted globally by RF (Figure 3) was similar to other studies [25,46,100], and so was the CLIMEX (Figure 4) [42,43]. Combining these models can offer more robust predictions, balancing the physiological realism of CLIMEX with the predictive power of statistical relationships of the correlative model.

### 4.3. Current and Future Habitat Suitability of Parthenium Weed in Bhutan

In Bhutan, habitat suitability was predicted solely based on the correlative RF model, as the CLIMEX model relied on its in-built climate datasets with 10–30′ resolution [101], which were coarse and not suitable for the mountainous country of Bhutan. The suitability estimated by the RF under current climate (Table 3) conditions was three times higher than that predicted by Thinley, et al. [48]. While their model indicated 8% of the area as suitable, our model suggested 23% (8919 km^2^) suitability. This discrepancy is attributed to variations in modeling approaches, resolution, and predictor variables. The current prediction showed that the southern belt adjoining India and the national highways of west–central (Punakha and Wangduephodrang), central (Trongsa and Zhemgang), and eastern Bhutan (Lhuentse, Mongar, and Trashigang) are more suitable to parthenium weed than other parts of the country (Figure 6, Supplementary Figures S2 and S3). These regions have sub-tropical and warm temperate type of climates with abundant rainfall and suitable temperatures during the summer [102], the season during which parthenium weed is found in the country. In India, Rameez, et al. [103] predicted a high suitability of parthenium weed in northeastern India, a region that shares a similar climate and topography with Bhutan. The location of predicted suitability along the road networks in Bhutan (Figure 6) is also indicative of roads as a major conduit through which parthenium weed is spread [24,104,105].

In the future, modelling showed a considerable variation across models, scenarios, and periods in parthenium weed suitability but was reduced generally from the 2021–2050, through the 2051–2080, and finally to the 2071–2100 period, compared to the current suitability (Table 3, Supplementary Table S6). This reduction was primarily attributed to projected decrease and variability in annual precipitation, which appeared to limit the weed’s habitat suitability under future climate conditions. This reduction in parthenium weed suitability contrasted with studies that generally indicated that climate change will enhance invasive species spread, as many reports suggest increased habitat suitability for invasive weeds [106,107,108]. However, our findings are consistent with those of Thinley, et al. [48], who projected a decline in suitability from 8% to 4% in Bhutan by 2070, as well as with similar studies in the region [23,58,103] and in other parts of the world [106,109,110]. It should be noted that while overall suitability for parthenium weed decreases across models, scenarios, and periods, a few areas, especially the two western districts of Paro and Thimphu, showed increased suitability in the future (Figure 6 and Figure 7, Supplementary Figures S2 and S3). Additionally, the effect of climate change on the habitat suitability of parthenium weed was evident, with projections showing a northward shift in its mean location by 2–76 km (Figure 10, Supplementary Figure S6 and Table S8) in the future, as observed in Pakistan [47]. This northward migration of invasive species is a well-documented consequence of climate change [111,112,113].

### 4.4. Potential Impact of Parthenium Weed on Agricultural Land in Bhutan

The assessment of the potential impact of the predicted suitability of parthenium weed on agricultural land in Bhutan showed that about 23% (2049 km^2^) (Table 4) of agricultural land in the country is currently at risk from parthenium weed. Parthenium weed intrusions into agricultural areas and crop loss are major concerns in affected countries like India and Australia. In India, parthenium weed caused a 40% yield loss in rice, 63% in tomato [*Lycopersicon esculentum* Mill.], and 90% in sorghum (*Sorhgum bicolor* (L.) Moench) [6]. In other countries like Ethiopia, sorghum loss of about 97% was reported [114], while in Pakistan, a density of 16 plants/m^−2^ caused a 46% yield loss in maize (*Zea mays* L.) [115]. Parthenium weed has also infested direct-seeded rice systems, with the potential to cause a ca. 33% yield loss [116]. The economic impact of such infestations would be considerable in Bhutan, given that agriculture is the mainstay of the country’s economy, with about 60% of the population dependent on agriculture. Predictions from GCMs indicate that the potential impact on agricultural land by parthenium weed in the future will decrease across various climate models, scenarios, and future periods (Table 4, Supplementary Figure S5 and Table S7). However, this decrease should be interpreted with caution, as climate models do not account for all factors influencing parthenium weed spread. While climate plays a major role in the weed’s growth, other environmental conditions such as soil moisture and land use change can also affect its distribution. Notably, irrigation has been observed to facilitate parthenium weed spread in regions like Pakistan [47], suggesting that areas with increased artificial moisture may remain vulnerable despite climate-driven declines in other regions.

### 4.5. Field Validation of RF Predictions

The field validation of RF was undertaken only for Bhutan, as it was the most feasible option practically. The validation took in April 2024, which was the onset of the parthenium weed season in Bhutan. Given time and resource constraints, the field work focused only on areas predicted as suitable. The field validation showed a high correspondence of parthenium weed occurrence, with suitability predictions for all the areas visited (n = 92) (Figure 11). This high correspondence further confirmed the robustness of the RF model and the reliability of its predictions for the globe and for Bhutan. The expert knowledge of the first author was also used to further validate the suitability predictions. The first author possesses more than 10 years of experience in the country working with the National Plant Protection Center in Bhutan looking after agricultural and environmental weeds and in Paro and Lhuentse districts as District Agriculture Officer.

### 4.6. Assumptions and Limitations

Despite the consistency of our results with previous research, there were necessary assumptions and limitations. The publicly available occurrence records of parthenium weed, in particular, with GBIF, is not without limitations. Sampling biases often arise due to the concentration of records in areas with greater research activity, easy accessibility, or locations of high economic importance, while remote or less studied areas may be underrepresented. Additionally, data quality issues, such as errors in georeferencing, duplicate records, and outdated information, can influence model predictions. To mitigate these limitations, careful data cleaning, filtering, and bias correction techniques (e.g., spatial thinning or background sampling) were applied to reduce the impact of sampling bias and improve model reliability. Additionally, data from other sources (Supplementary Table S1) were incorporated to reduce data errors and improve coverage. Despite these efforts, residual biases may have been still existed and influenced the model outcomes.

Irrigation was not included in the model due to the lack of global and Bhutan-specific data describing irrigation under current and future scenarios. The RF model was also trained globally using WorldClim [34] data at a 30 s (ca. 1 km at the equator) resolution, while predictions for parthenium weed distribution in Bhutan were made using downscaled GCMs at a finer 250 m resolution. The impact of these resolution differences on the model’s suitability predictions in Bhutan remains unclear. Additionally, while downscaling enhances spatial resolution, it is not without limitations; the downscaling process itself can introduce uncertainties, and inherent biases in GCMs cannot be fully corrected through downscaling alone. Moreover, projections of future climate suitability depend on various scenarios, including greenhouse gas emissions, land-use changes, and socio-economic factors. The inherent uncertainty of these variables contributes to the unpredictability of model outputs.

Regarding model flexibility, the RF model offers greater adaptability by leveraging global species distribution data, whereas the CLIMEX model explicitly models the response to climate and weather responses, which requires a detailed understanding of the species’ ecology. While parthenium weed has been widely studied, this mechanistic approach may be limited by gaps in ecological knowledge and specific in situ conditions. Thus, while these models provide valuable insights, caution is needed in interpreting predictions, as multiple factors may affect their accuracy.

Future studies should focus on integrating mechanistic and correlative models or developing a hybrid model for more robust predictions and a deeper understanding of ecological dynamics, predict the suitability using models specifically trained for Bhutan and incorporate a wider range of modeling approaches, and integrate 250 m downscaled bioclimatic variables into the CLIMEX model to enable its application within Bhutan and facilitate more precise comparisons between correlative models and CLIMEX for enhanced accuracy and insight.

## 5. Conclusions

Based on AUC, TSS, sensitivity, and specificity, RF was identified as the best model. The annual mean temperature (bio1) was the most important predictor for parthenium weed habitat suitability. Globally, RF predicted 7 million km^2^ (2%) of the total land mass as suitable to parthenium weed, while CLIMEX predicted 20 million km^2^ (13%). However, RF identified 67 countries not predicted as suitable by CLIMEX, while CLIMEX predicted 20 countries not predicted as suitable by RF. RF predicted actual distributions, including temperate regions, while CLIMEX predicted suitability ranges and was more focused on tropical and sub-tropical regions. Nonetheless, RF provided an essential complement to broader predictions of CLIMEX, offering granular insights for more targeted ecological management and conservation planning.

For Bhutan, RF predicted that 23% (8919 km^2^) of the country’s total area is currently suitable for parthenium weed, with high vulnerability in southern, western, and eastern districts, especially along national highways. Future predictions by 10 GCMs under different climate scenarios (SSP126, SSP245, SSP370, SSP585) and time periods (2021–2050, 2051–2080, 2071–2100) showed variability in suitability levels. However, all models predicted an overall decline in suitability over time. A northward shift in suitable regions, ranging from 2 km (INM-CM5-0) to 76 km (MPI-ESM1-2-LR), is expected, with areas like Paro, Thimphu, and the southeast becoming more vulnerable. Additionally, 23% (2049 km^2^) of agricultural land is currently at risk of being under parthenium weed.

Since correlative and mechanistic models are based on different concepts yielding different niches and geographic extents, integrating them will provide a more comprehensive assessment. In Bhutan, although future predictions indicate a decline in suitability, high suitability is predicted under the current climate. Therefore, parthenium weed is expected to exert economic pressure and increase labor demands on Bhutan’s agricultural sector. Given that parthenium weed is in its early invasion stages, early interventions such as early detection and control of infested areas, regular survey and monitoring in high suitability sites, and creating public awareness are proposed to mitigate the expected negative impacts on agriculture, the environment, and public health in Bhutan.

## Figures and Tables

**Table 1:** Environmental predictors used for parthenium weed habitat suitability modelling globally and in Bhutan. These variables were selected after testing for multicollinearity, literature review, and ecological relevance.

Variable	Full Name	Unit
Bio1	Annual mean temperature	°C
Bio2	Mean diurnal range	°C
Bio4	Temperature seasonality	°C
Bio8	Mean temperature of wettest quarter	°C
Bio9	Mean temperature of driest quarter	°C
Bio12	Annual precipitation	mm
Bio14	Precipitation of driest month	mm
Bio15	Precipitation seasonality	Dimensionless
Bio18	Precipitation of the warmest quarter	mm
Bio19	Precipitation of the coldest quarter	mm

**Table 2:** Statistical evaluation of models trained on the global distribution of parthenium weed with presence-pseudo-absence data (occurrence = 7935; pseudo-absence = 7828), with five-fold cross-validation, repeated five times. Model performances were evaluated using the true skill statistic, area under the receiver operating characteristic curve, sensitivity (true positive rate), and specificity (true negative rate) of the individual (RF, GBM, GAM, CLIMEX) and ensemble (mean) of the algorithms.

Model	Threshold	Sensitivity	Specificity	TSS	AUC
RF	0.588	0.86	0.82	0.68	0.92
GBM	0.513	0.78	0.73	0.51	0.82
GAM	0.532	0.80	0.69	0.49	0.81
Ensemble	0.541	0.81	0.79	0.60	0.88
CLIMEX	0.26	0.61	0.70	0.30	0.69

Abbreviations: RF, random forest; GBM, generalized boosted model; GAM, generalized additive model.

**Table 3:** Predicted area of suitability for parthenium weed in Bhutan under four shared-socio economic pathways (SSPs)—SSP126, SSP245, SSP370, and SSP585—in the 2051–2080 period based on 10 general circulation models in Bhutan.

Predicted Current Suitability: 8919 km^2^ (23%)				
	Predicted Area of Future Suitability (km^2^)			
GCMs Model	SSP126	SSP245	SSP370	SSP585
ACCESS-CM2	5019	2849	2464	3661
	(56%)	(32%)	(28%)	(41%)
CNRM-CM6-1	3222	1755	742	652
	(36%)	(20%)	(8%)	(7%)
CNRM-ESM2-1	2984	1455	2518	1410
	(34%)	(16%)	(28%)	(16%)
INM-CM4-8	2942	3347	3039	2742
	(33%)	(38%)	(34%)	(31%)
INM-CM5-0	2972	1976	1111	1489
	(33%)	(22%)	(12%)	(17%)
MIROC6	3898	3047	1429	3348
	(44%)	(34%)	(16%)	(38%)
MIROC-ES2L	2641	1777	1466	776
	(30%)	(20%)	(16%)	(9%)
MPI-ESM1-2-LR	2643	1180	692	557
	(30%)	(13%)	(8%)	(6%)
MRI-ESM2-0	4224	3027	1874	2881
	(47%)	(34%)	(21%)	(32)
NorESM2-MM	2835	2344	4866	1124
	(32%)	(26%)	(55%)	(13%)

**Table 4:** Area of predicted suitability for parthenium weed on agricultural land under the current climate (1986–2015) and in the future under four shared-socio economic pathways (SSPs)—SSP126, SSP245, SSP370, and SSP585—in the 2051–2080 period based on 10 GCMs in Bhutan.

	Area (km^2^) of Agricultural Land at Risk			
Current: 2049 (23%)				
GCMs Model	SSP126	SSP245	SSP370	SSP585
ACCESS-CM2	1420	816	690	994
	(28%)	(29%)	(28%)	(27%)
CNRM-ESM2-1	966	492	784	442
	(32%)	(34%)	(31%)	(31%)
CNRM-CM6-1	977	564	276	218
	(30%)	(32%)	(37%)	(34%)
INM-CM4-8	1003	1105	996	936
	(34%)	(33%)	(33%)	(34%)
INM-CM5-0	1062	1299	695	743
	(33%)	(31%)	(33%)	(29%)
MIROC6	1217	988	530	1014
	(31%)	(32%)	(37%)	(30%)
MIROC-ES2L	873	536	450	230
	(33%)	(30%)	(31%)	(30%)
MPI-ESM1-2-LR	953	475	325	209
	(36%)	(40%)	(47)	(37)
MRI-ESM2-0	1287	968	542	792
	(31%)	(32%)	(29%)	(28%)
NorESM2-MM	922	774	1381	399
	(33%)	(33)	(28%)	(36%)

## Data Availability

Data are contained within the article and Supplementary Materials.

## Supplementary Materials

The following supporting information can be downloaded at: https://www.mdpi.com/article/10.3390/plants14010083/s1, Figure S1: Global distribution of parthenium weed (black points) showing native countries (green), invaded countries (red), and countries with transient populations (orange). Source: GBIF (2024); Figure S2: Map of Bhutan showing the 20 districts along with the road networks. Figure S3: Predictive maps of predicted suitability of parthenium weed in Bhutan in the future. From the top: row 1–3, SSP126 in the 2021–2050, 2051–2080, and 2071–2100 periods; row 4–6, SSP245 in the 2021–2050, 2051–2080, and 2071–2100 periods; row 7–9, SSP370 in the 2021–2050, 2051–2080, and 2071–2100 periods; and row 10–12, SSP585 in the 2021–2050, 2051–2080, and 2071–2100 periods. The suitability is indicated by the green color, with higher intensity indicating higher suitability; Figure S4: Maps of changes in predicted suitability of parthenium weed in Bhutan in the future. From the top: row 1–3, SSP126 in the 2021–2050, 2051–2080, and 2071–2100 periods; row 4–6, SSP245 in the 2021–2050, 2051–2080, and 2071–2100 periods; row 7–9, SSP370 in the 2021–2050, 2051–2080, and 2071–2100 periods; and row 10–12, SSP585 in the 2021–2050, 2051–2080, and 2071–2100 periods. Gray regions represent areas where the weed’s distribution is predicted to lose, while green and red regions highlight areas of no change and gain in distribution, respectively. Notably, new areas of high suitability were predicted along highways in Paro and Thimphu; Figure S5: Maps of predicted agricultural land suitability (red) to parthenium weed against the total agricultural land available (green) in the country. From the top: row 1–3, SSP126 in the 2021–2050, 2051–2080, and 2071–2100 periods; row 4–6, SSP245 in the 2021–2050, 2051–2080, and 2071–2100 periods; row 7–9, SSP370 in the 2021–2050, 2051–2080, and 2071–2100 periods; and row 10–12, SSP585 in the 2021–2050, 2051–2080, and 2071–2100 periods; Figure S6: The standard deviational ellipse (SDE) and the mean location of parthenium weed distribution under the current and future climate. From the top: row 1–3, SSP126 in the 2021–2050, 2051–2080, and 2071–2100 periods; row 4–6, SSP245 in the 2021–2050, 2051–2080, and 2071–2100 periods; row 7–9, SSP370 in the 2021–2050, 2051–2080, and 2071–2100 periods; and row 10–12, SSP585 in the 2021–2050, 2051–2080, and 2071–2100 periods. Different colors represent the ellipses for various general circulation models (GCMs), with each model’s mean location marked by distinct symbols: Table S1: Sources of parthenium weed occurrence data; Table S2: The multicollinearity of 19 bioclimatic variables was tested using the vifcor at threshold = 0.7 and the Spearman method; Table S3: Review of bioclimatic variables used in parthenium weed modelling globally; Table S4: Model parameter setting used in CLIMEX; Table S5: List of countries predicted suitable by random forest (RF), which are not in CLIMEX, and vice-versa. RF identified 67 unique countries, while CLIMEX identified 20 unique countries not common to each other; Table S6: Predicted future suitability of parthenium weed under four shared socio-economic pathways (SSP), SSP126, SSP245, SSP370, and SSP585, in the three periods, 2021–2040, 2041–2080, and 2071–2100, in Bhutan. Figures in the parenthesis indicate suitability in percentages compared to the current suitability; Table S7: Predicted suitability of agricultural land to parthenium weed in the future under the four scenarios, SSP126, SSP245, SSP370, and SSP585, and the three periods, 2021–2050, 2051–2080, and 2071–2100, in Bhutan. Figures in the brackets indicate percentages of the predicted agricultural land suitability from the total suitability predicted by each general circulation model; Table S8: Shift in the mean location (km) of parthenium weed distribution in the future under four scenarios, SSP126, SSP245, SSP370, and SSP585, and in three periods, 2021–2050, 2051–2080, and 2071–2100.
