# Supplementary material for: Comparative Analysis of Mechanistic and Correlative Models for Global and Bhutan-Specific Suitability of Parthenium Weed and Vulnerability of Agriculture in Bhutan
Source: Plants (Basel). 2024 Dec 30;14(1):83. doi: 10.3390/plants14010083 (PMC11722948; doi:10.3390/plants14010083)
Supplement: Supplementary file 1 [file plants-14-00083-s001.zip › plants-3362206-supplementary.pdf]

Table S1. Sources of parthenium weed occurrence data.

Data	Count	Reference
Australasian Virtual Herbarium	3676	https://avh.chah.org.au/
Bhutan	335	Chhogyel *et al.* (2021), Dorji *et al.* (2021)
China	171	Mao (2022), Mao (2018)
Nepal	1093	Shrestha and Shrestha (2020)
Pakistan	221	Shabbir *et al.* (2012)
Global Biodiversity Information Facility (GBIF)	27968	https://www.gbif.org/, Accessed on 14/05/2024
Total	33464	

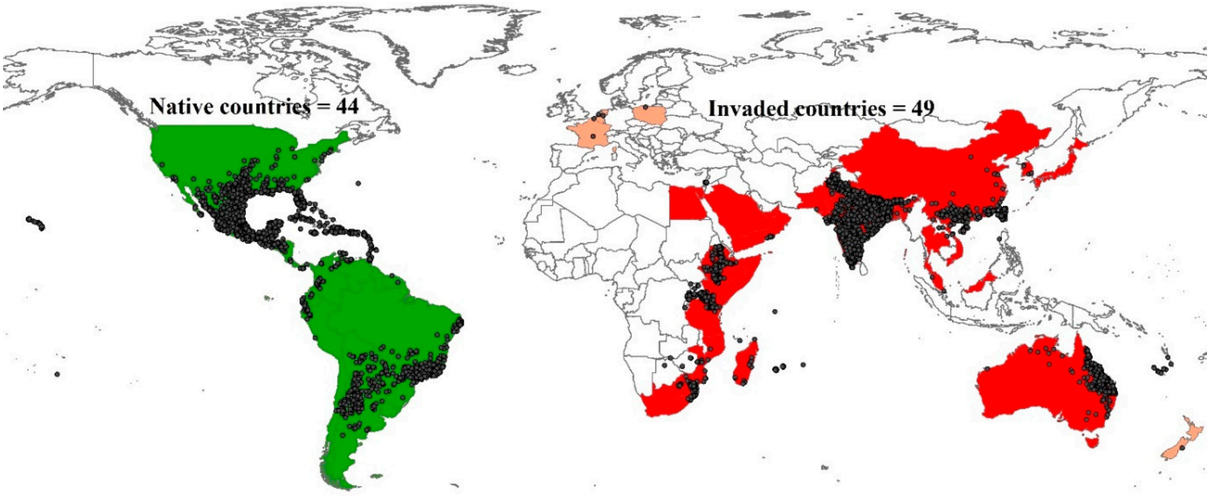

Figure S1. Global distribution of parthenium weed (black points) showing native countries (green), invaded countries (red), and countries with transient populations (orange). Source: GBIF (2024).

Table S2. The multicollinearity of 19 bioclimatic variables was tested using the vifcor at threshold = 0.7 and Spearman method.

Variables	VIF
bio2	1.8429
bio8	2.5865
bio9	2.1239
bio15	1.2786
bio18	1.5151
bio19	1.5467

Table S3. Review of bioclimatic variables used in parthenium weed modelling globally.

Bioclimatic variables	References
Bio1, Bio3, Bio12, Bio14, Bio15	Lamsal *et al.* (2018)
Bio3, Bio8, Bio14, Bio15, Bio19	Chhogyel *et al.* (2021)
Bio1, Bio2, Bio3, bio12, Bio13, Bio14	Adhikari *et al.* (2023)
Bio2, Bio3, Bio12, Bio14, Bio15, Bio17	Maharjan *et al.* (2019)

Bio2, Bio3, Bio6, Bio8, Bio15, Bio18, Bio19	Dorji *et al.* (2021)
Bio2, Bio3, Bio9, Bio14, Bio15, Bio17, Bio18	Shrestha *et al.* (2018)
Bio1, Bio2, Bio3, Bio4, Bio5, Bio12, Bio15, Bio19	Ruheili *et al.* (2022)
Bio1, Bio4, Bio6, Bio7, Bio10, Bio14, Bio16, Bio17	Masum *et al.* (2022)
Bio1, Bio2, Bio3, Bio4, Bio5, Bio12, Bio14, Bio15, Bio19	Mushtaq *et al.* (2021)
Bio1, Bio2, Bio3, Bio4, Bio5, Bio6, Bio7, Bio8, Bio9, Bio10, Bio11	Panetta and Mitchell (1991)
Bio1, Bio2, Bio3, Bio4, Bio5, Bio12, Bio14, Bio15, Bio18, Bio19	Ahmad *et al.* (2019)
Bio1, Bio3, Bio5, Bio12, Bio13, Bio14, Bio15, Bio16, Bio18, Bio19	Thinley *et al.* (2019)
All the 19 bioclimatic variables	Mainali *et al.* (2015); Barasa (2022)

Table S4. Model parameter setting used in CLIMEX.

Index	Parameter	Description	Values	Units
Temperature	DV0	Limiting low temperature	5	℃
	DV1	Lower optimum temperature	25	℃
	DV2	Upper optimum temperature	30	℃
	DV3	Limiting high temperature	42	℃
Moisture	SM0	Limiting low soil moisture	0.08	
	SM1	Lower optimum soil moisture	0.2	
	SM2	Upper optimum soil moisture	0.6	
	SM3	Limiting high soil moisture	1.6	
Cold stress	TTCS	Temperature threshold	4	℃
	THCS	Stress accumulation rate	-0.001	$Week^{-1}$
	DTCS	Degree-day threshold	12	day
	DHCS	Degree-day stress rate	-0.0001	$Week^{-1}$
Heat stress	TTHS	Temperature threshold	42	℃
	THHS	Stress accumulation rate	0.001	$Week^{-1}$
Dry stress	SMDS	Wet stress threshold	0.07	
	HDS	Stress accumulation rate	-0.001	$Week^{-1}$
Wet stress	SMWS	Wet stress threshold	2.3	
	HWS	Stress accumulation rate	0.002	$Week^{-1}$
Hot-dry stress	TTHD	Got-dry temperature threshold	36	
	MTHD	Hot-dry moisture threshold	0.2	$Week^{-1}$
	PHD	Stress accumulation rate	0.001	
Annual heat sum	PDD	Degree-day threshold	2000	℃ Day

Table S5. List of countries predicted suitable by random forest (RF), which are not in CLIMEX, and vice-versa. RF identified 67 unique countries, while CLIMEX identified 20 unique countries not common to each other.

Unique countries identified by RF	Unique countries identified by CLIMEX
Aruba	Afghanistan
Austria	Albania
Belgium	Azerbaijan
Bermuda	Bosnia and Herzegovina
Bhutan	Central African Republic
Burkina Faso	Croatia
Canada	Djibouti
Chad	Equatorial Guinea
Chile	Gabon
Cook Islands	Georgia
Cyprus	Guantanamo Bay Naval Base
Czech Republic	Liberia
Denmark	Monaco
Dominica	Montenegro
El Salvador	Panama
Estonia	Sierra Leone
Finland	Sudan
French Polynesia	São Tomé and Príncipe
French Southern and Antarctic Lands	Uruguay
Germany	Vatican City
Gibraltar	
Greece	
Grenada	
Guam	
Hong Kong	
Iraq	
Israel	
Japan	
Jordan	
Kiribati	
Latvia	
Lebanon	
Libya	
Lithuania	
Macau	
Malaysia	
Maldives	
Mali	
Malta	
Marshall Islands	
Montserrat	
Niue	
Norfolk Island	
North Korea	
Northern Mariana Islands	
Norway	

Oman	
Palestine	
Pitcairn Islands	
Poland	
Saint Kitts and Nevis	
Saint Lucia	
Saint Vincent and the Grenadines	
Saint-BarthÃ©lemy	
Senegal	
Seychelles	
Singapore	
South Korea	
Sweden	
Switzerland	
Syria	
Tonga	
Tuvalu	
Ukraine	
United Arab Emirates	
United Kingdom	
United States Minor Outlying Islands	

Table S6. Predicted future suitability of parthenium weed under four shared socio-economic pathways (SSP), SSP126, SSP245, SSP370, and SSP585 in the three periods, 2021-2040, 2041-2080, and 2071-2100 in Bhutan. Figures in the parenthesis indicate suitability in percentages compared to the current suitability.

	Predicted future suitability areas (km^2)			
	2021-2050			
**Model**	**SSP126**	**SSP245**	**SSP370**	**SSP585**
ACCESS-CM2	4034.12 (45.23)	3913.94 (43.88)	3096.75 (34.72)	3505.12 (39.30)
CNRM-CM6-1	2401.31 (26.92)	2645.50 (29.66)	2221.94 (24.91)	1578.50 (17.70)
CNRM-ESM2-1	2389.00 (26.79)	4132.69 (46.34)	2864.19 (32.11)	2137.00 (23.96)
INM-CM4-8	3319.38 (37.22)	3035.94 (34.04)	3375.38 (37.85)	3354.31 (37.61)
INM-CM5-0	2192.50 (24.58)	2283.75 (25.61)	1989.12 (22.30)	2561.81 (28.72)
MIROC6	4770.31 (53.49)	3551.06 (39.82)	2364.81 (26.51)	3567.50 (40.00)
MIROC-ES2L	2872.81 (32.21)	2080.12 (23.32)	1739.44 (19.50)	2428.88 (27.23)
MPI-ESM1-2-LR	2066.75 (23.17)	2739.62 (30.72)	2474.19 (27.74)	2290.81 (25.69)
MRI-ESM2-0	2973.50 (33.34)	3299.81 (37.00)	3830.25 (42.95)	2520.81 (28.26)
NorESM2-MM	3095.06 (34.70)	4891.56 (54.85)	2486.12 (27.88)	5365.81 (60.16)
	2051-2080			
ACCESS-CM2	5019.50 (56.28)	2849.06 (31.94)	2464.06 (27.63)	3661.00 (41.05)
CNRM-CM6-1	3221.62 (36.12)	1754.88 (19.68)	741.50 (8.31)	651.94 (7.31)
CNRM-ESM2-1	2984.25 (33.46)	1454.69 (16.31)	2517.81 (28.23)	1409.69 (15.81)
INM-CM4-8	2942.00 (32.99)	3346.56 (37.52)	3039.19 (34.08)	2741.81 (30.74)
INM-CM5-0	2971.81 (33.32)	1976.00 (22.16)	1110.88 (12.46)	1488.75 (16.69)
MIROC6	3898.31 (43.71)	3047.25 (34.17)	1428.56 (16.02)	3347.81 (37.54)
MIROC-ES2L	2641.38 (29.62)	1776.50 (19.92)	1466.44 (16.44)	775.50 (8.70)

MPI-ESM1-2-LR	2642.88 (29.63)	1180.12 (13.23)	692.00 (7.76)	556.56 (6.24)
MRI-ESM2-0	4224.06 (47.36)	3026.94 (33.94)	1874.12 (21.01)	2880.62 (32.30)
NorESM2-MM	2834.56 (31.78)	2344.38 (26.29)	4865.56 (54.55)	1124.06 (12.60)
	**2071-2100**			
ACCESS-CM2	4627.75 (51.89)	2720.69 (30.51)	2531.88 (28.39)	4351.94 (48.80)
CNRM-CM6-1	2572.44 (28.84)	1481.44 (16.61)	556.31 (6.24)	1060.62 (11.89)
CNRM-ESM2-1	2131.75 (23.90)	776.25 (8.70)	1112.19 (12.47)	900.62 (10.10)
INM-CM4-8	3205.56 (35.94)	4139.75 (46.42)	2136.25 (23.95)	2587.56 (29.01)
INM-CM5-0	3181.25 (35.67)	1761.12 (19.75)	1120.94 (12.57)	2159.31 (24.21)
MIROC6	5408.25 (60.64)	2599.06 (29.14)	998.69 (11.20)	2369.19 (26.56)
MIROC-ES2L	4217.88 (47.29)	2676.62 (30.01)	467.19 (5.24)	1206.75 (13.53)
MPI-ESM1-2-LR	2032.25 (22.79)	705.69 (7.91)	373.06 (4.18)	143.06 (1.60)
MRI-ESM2-0	4473.25 (50.16)	3677.38 (41.23)	2599.19 (29.14)	3679.88 (41.26)
NorESM2-MM	3628.50 (40.68)	2164.50 (24.27)	3767.00 (42.24)	2193.88 (24.60)

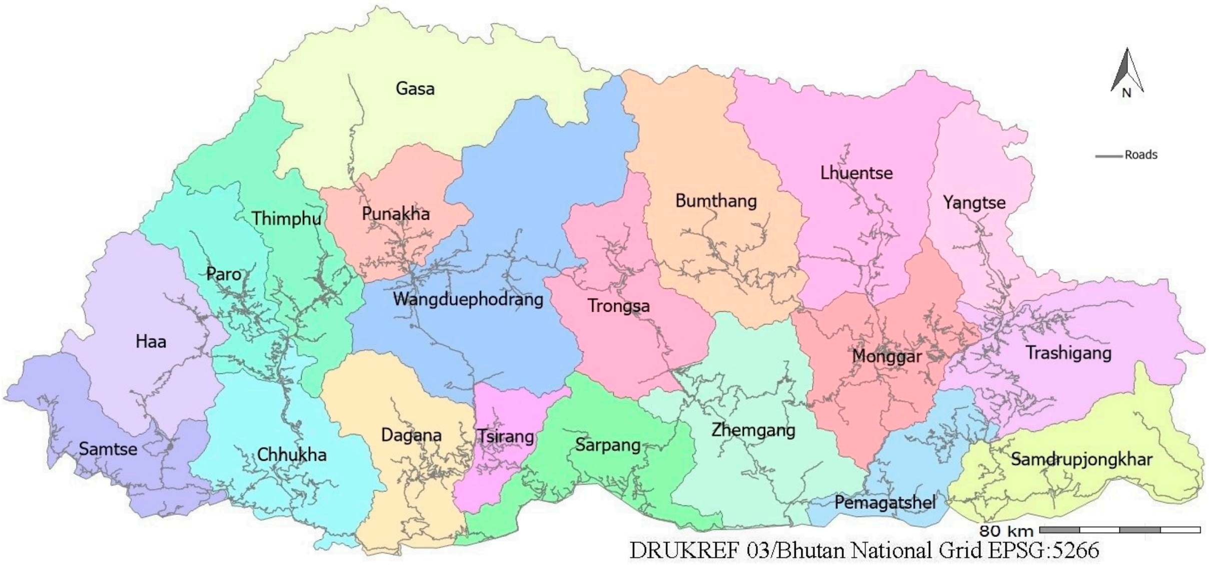

Figure S2. Map of Bhutan showing the 20 Districts along with the road networks.

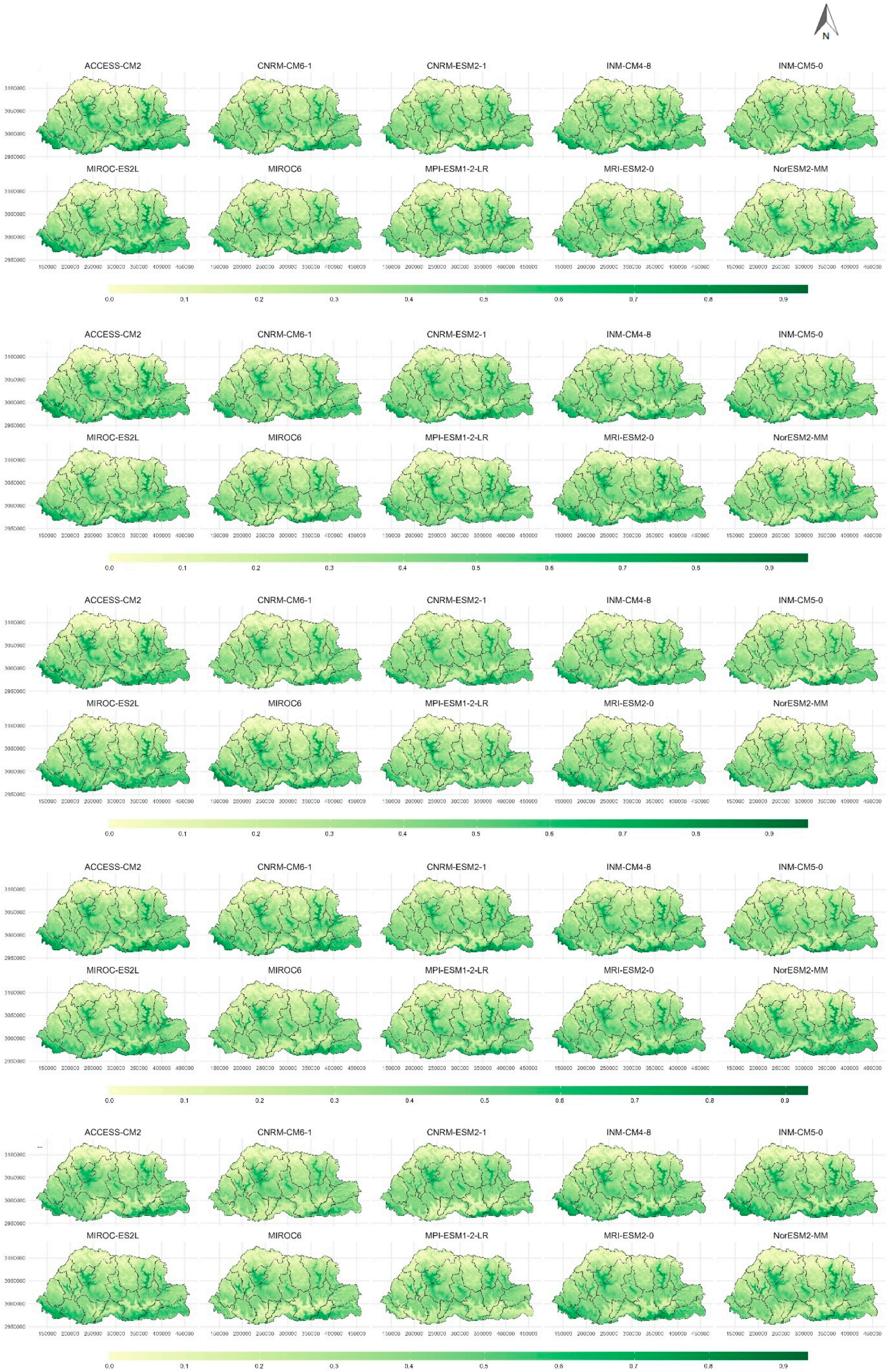
N
ACCESS-CM2
CNRM-CM6-1
CNRM-ESM2-1
INM-CM4-8
INM-CM5-0
MIROC-ES2L
MIROC6
MPI-ESM1-2-LR
MRI-ESM2-0
NorESM2-MM
0.0 0.1 0.2 0.3 0.4 0.5 0.6 0.7 0.8 0.9

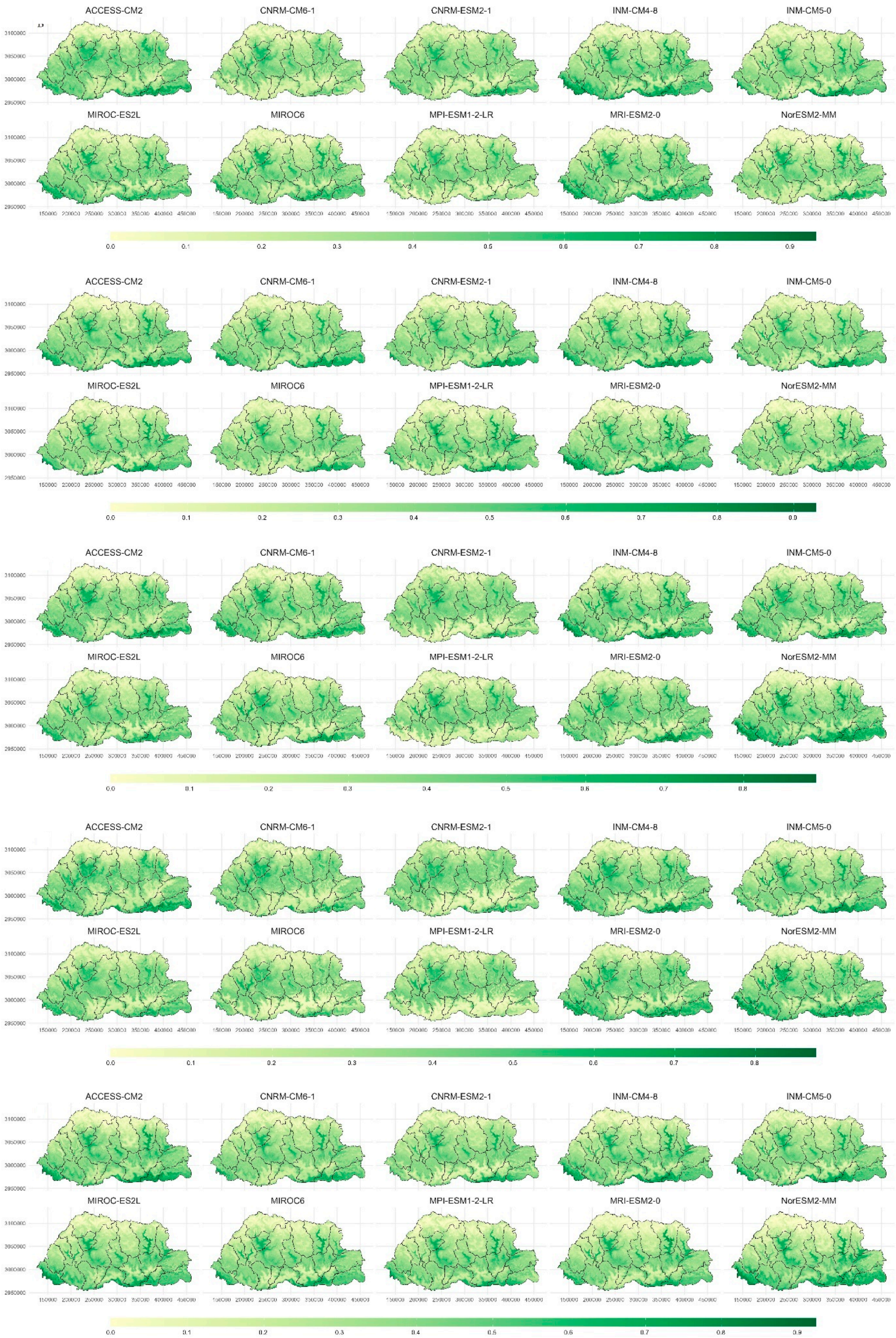
ACCESS-CM2
CNRM-CM6-1
CNRM-ESM2-1
INM-CM4-8
INM-CM5-0
MIROC-ES2L
MIROC6
MPI-ESM1-2-LR
MRI-ESM2-0
NorESM2-MM
3100000
3050000
3000000
2950000
150000 200000 250000 300000 350000 400000 450000
0.0 0.1 0.2 0.3 0.4 0.5 0.6 0.7 0.8 0.9
ACCESS-CM2
CNRM-CM6-1
CNRM-ESM2-1
INM-CM4-8
INM-CM5-0
MIROC-ES2L
MIROC6
MPI-ESM1-2-LR
MRI-ESM2-0
NorESM2-MM
0.0 0.1 0.2 0.3 0.4 0.5 0.6 0.7 0.8 0.9
ACCESS-CM2
CNRM-CM6-1
CNRM-ESM2-1
INM-CM4-8
INM-CM5-0
MIROC-ES2L
MIROC6
MPI-ESM1-2-LR
MRI-ESM2-0
NorESM2-MM
0.0 0.1 0.2 0.3 0.4 0.5 0.6 0.7 0.8
ACCESS-CM2
CNRM-CM6-1
CNRM-ESM2-1
INM-CM4-8
INM-CM5-0
MIROC-ES2L
MIROC6
MPI-ESM1-2-LR
MRI-ESM2-0
NorESM2-MM
0.0 0.1 0.2 0.3 0.4 0.5 0.6 0.7 0.8
ACCESS-CM2
CNRM-CM6-1
CNRM-ESM2-1
INM-CM4-8
INM-CM5-0
MIROC-ES2L
MIROC6
MPI-ESM1-2-LR
MRI-ESM2-0
NorESM2-MM
0.0 0.1 0.2 0.3 0.4 0.5 0.6 0.7 0.8 0.9

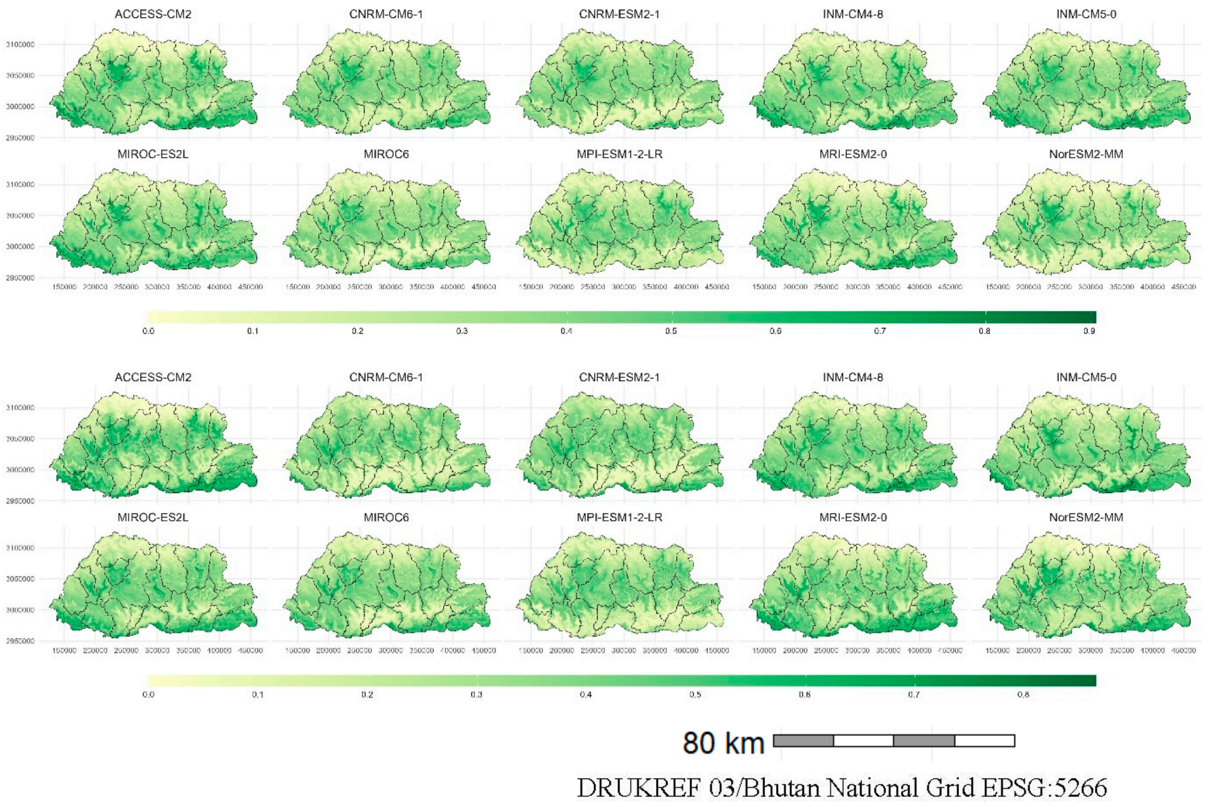

Figure S3. Predictive maps of predicted suitability of parthenium weed in Bhutan in the future. From the top: row 1 – 3, SSP126 in 2021-2050, 2051-2080, 2071-2100 periods; row 4 – 6, SSP245 in 2021-2050, 2051-2080, 2071-2100 periods; row 7 – 9, SSP370 in 2021-2050, 2051-2080, 2071-2100 periods; and row 10 – 12, SSP585 in 2021-2050, 2051-2080, 2071-2100 periods. The suitability is indicated by the green colour with higher intensity indicating higher suitability.

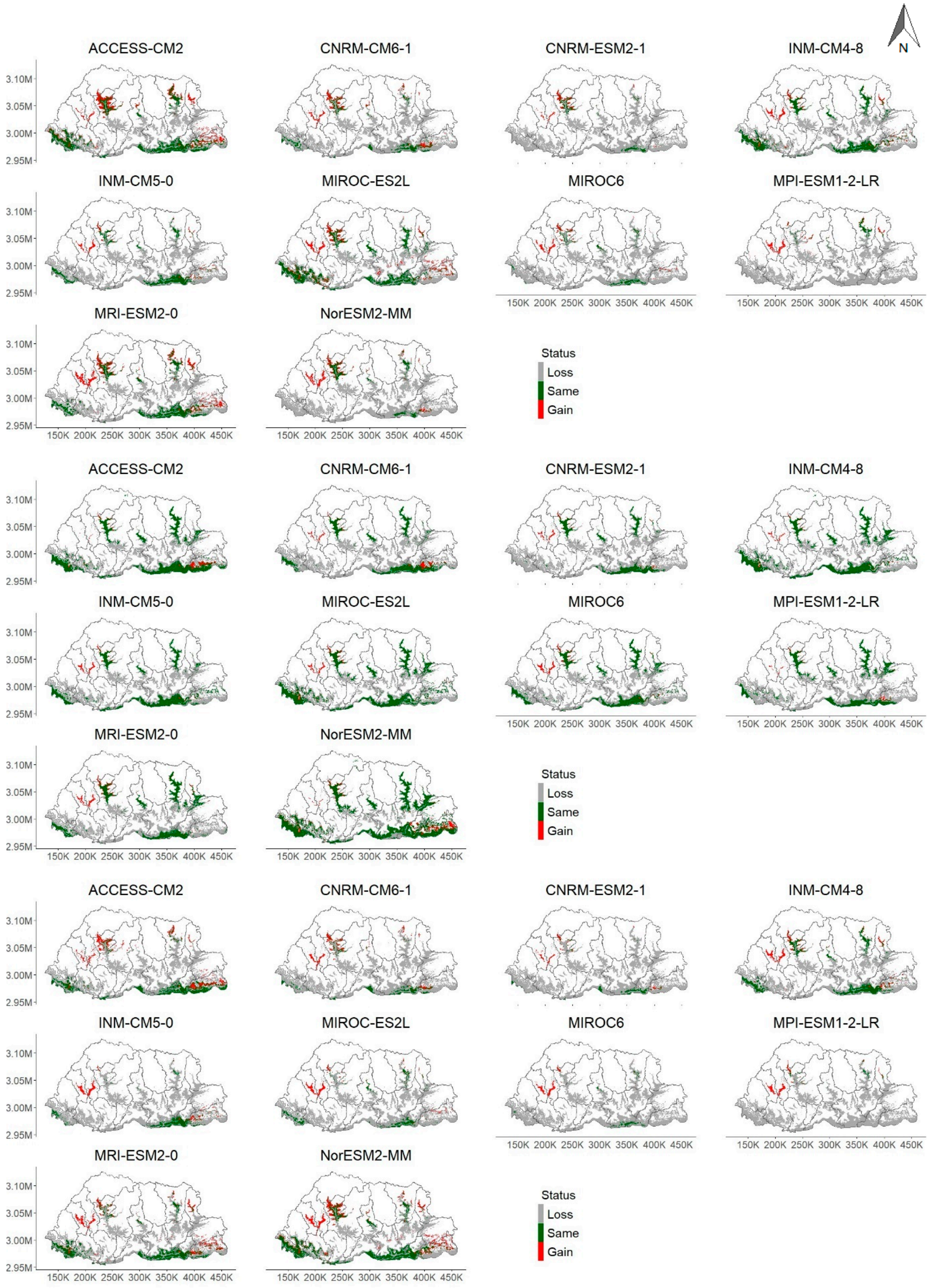
N
ACCESS-CM2
CNRM-CM6-1
CNRM-ESM2-1
INM-CM4-8
INM-CM5-0
MIROC-ES2L
MIROC6
MPI-ESM1-2-LR
MRI-ESM2-0
NorESM2-MM
3.10M
3.05M
3.00M
2.95M
150K 200K 250K 300K 350K 400K 450K
Status
Loss
Same
Gain
ACCESS-CM2
CNRM-CM6-1
CNRM-ESM2-1
INM-CM4-8
INM-CM5-0
MIROC-ES2L
MIROC6
MPI-ESM1-2-LR
MRI-ESM2-0
NorESM2-MM
3.10M
3.05M
3.00M
2.95M
150K 200K 250K 300K 350K 400K 450K
Status
Loss
Same
Gain
ACCESS-CM2
CNRM-CM6-1
CNRM-ESM2-1
INM-CM4-8
INM-CM5-0
MIROC-ES2L
MIROC6
MPI-ESM1-2-LR
MRI-ESM2-0
NorESM2-MM
3.10M
3.05M
3.00M
2.95M
150K 200K 250K 300K 350K 400K 450K
Status
Loss
Same
Gain

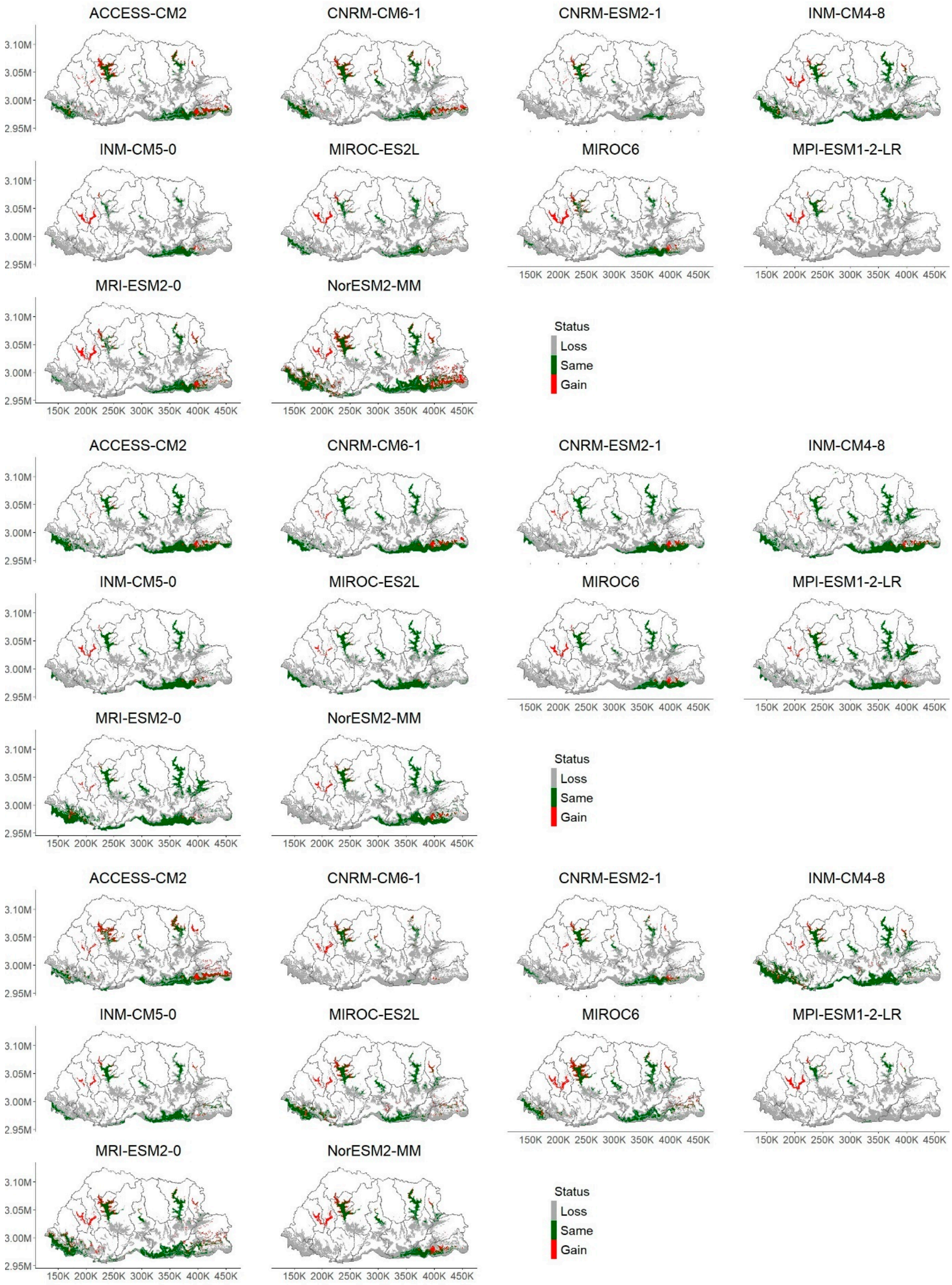
ACCESS-CM2
CNRM-CM6-1
CNRM-ESM2-1
INM-CM4-8
INM-CM5-0
MIROC-ES2L
MIROC6
MPI-ESM1-2-LR
MRI-ESM2-0
NorESM2-MM
3.10M
3.05M
3.00M
2.95M
150K 200K 250K 300K 350K 400K 450K
Status
Loss
Same
Gain
ACCESS-CM2
CNRM-CM6-1
CNRM-ESM2-1
INM-CM4-8
INM-CM5-0
MIROC-ES2L
MIROC6
MPI-ESM1-2-LR
MRI-ESM2-0
NorESM2-MM
Status
Loss
Same
Gain
ACCESS-CM2
CNRM-CM6-1
CNRM-ESM2-1
INM-CM4-8
INM-CM5-0
MIROC-ES2L
MIROC6
MPI-ESM1-2-LR
MRI-ESM2-0
NorESM2-MM
Status
Loss
Same
Gain

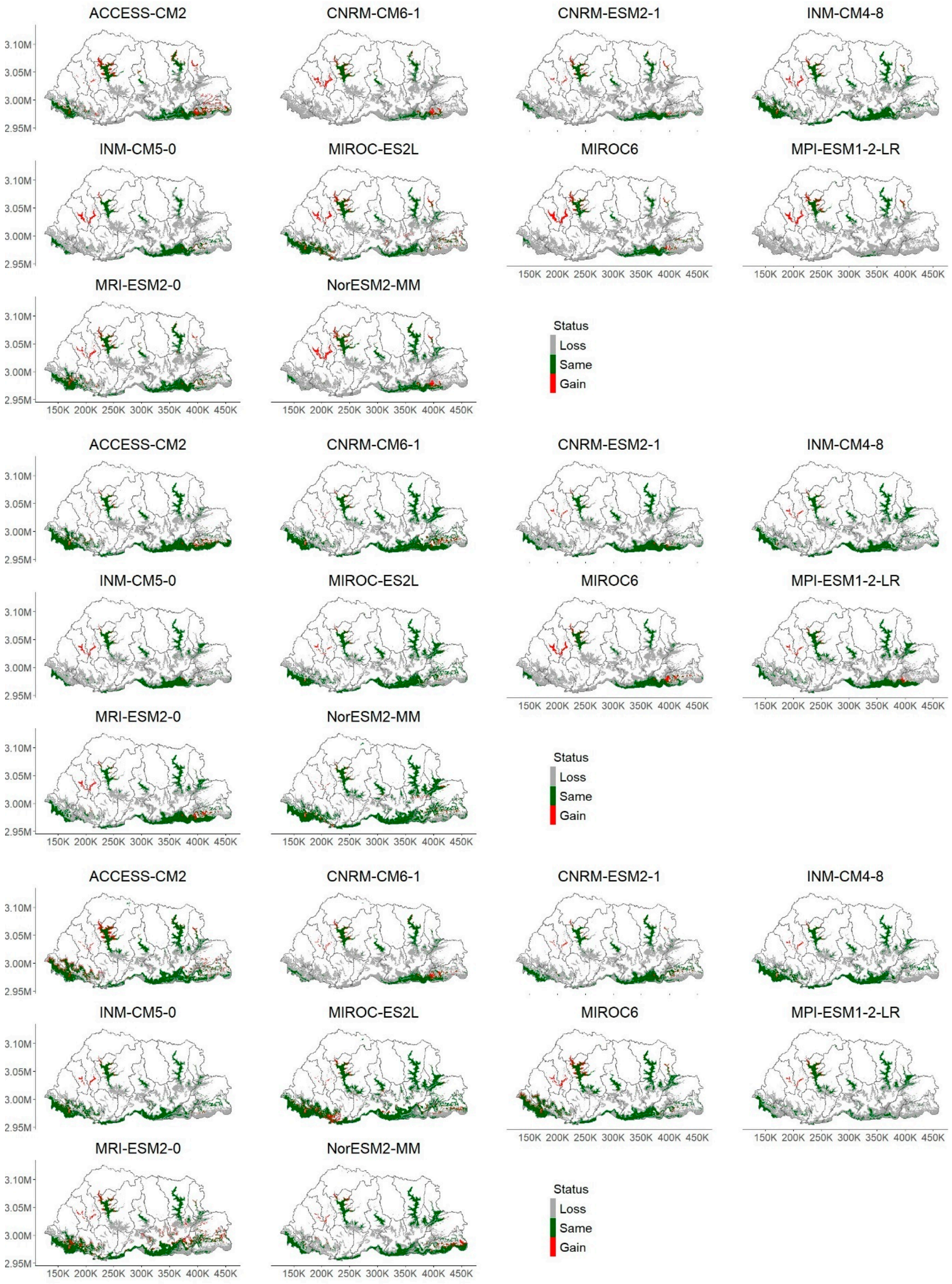
ACCESS-CM2
CNRM-CM6-1
CNRM-ESM2-1
INM-CM4-8
INM-CM5-0
MIROC-ES2L
MIROC6
MPI-ESM1-2-LR
MRI-ESM2-0
NorESM2-MM
3.10M
3.05M
3.00M
2.95M
150K 200K 250K 300K 350K 400K 450K
Status
Loss
Same
Gain
ACCESS-CM2
CNRM-CM6-1
CNRM-ESM2-1
INM-CM4-8
INM-CM5-0
MIROC-ES2L
MIROC6
MPI-ESM1-2-LR
MRI-ESM2-0
NorESM2-MM
3.10M
3.05M
3.00M
2.95M
150K 200K 250K 300K 350K 400K 450K
Status
Loss
Same
Gain
ACCESS-CM2
CNRM-CM6-1
CNRM-ESM2-1
INM-CM4-8
INM-CM5-0
MIROC-ES2L
MIROC6
MPI-ESM1-2-LR
MRI-ESM2-0
NorESM2-MM
3.10M
3.05M
3.00M
2.95M
150K 200K 250K 300K 350K 400K 450K
Status
Loss
Same
Gain

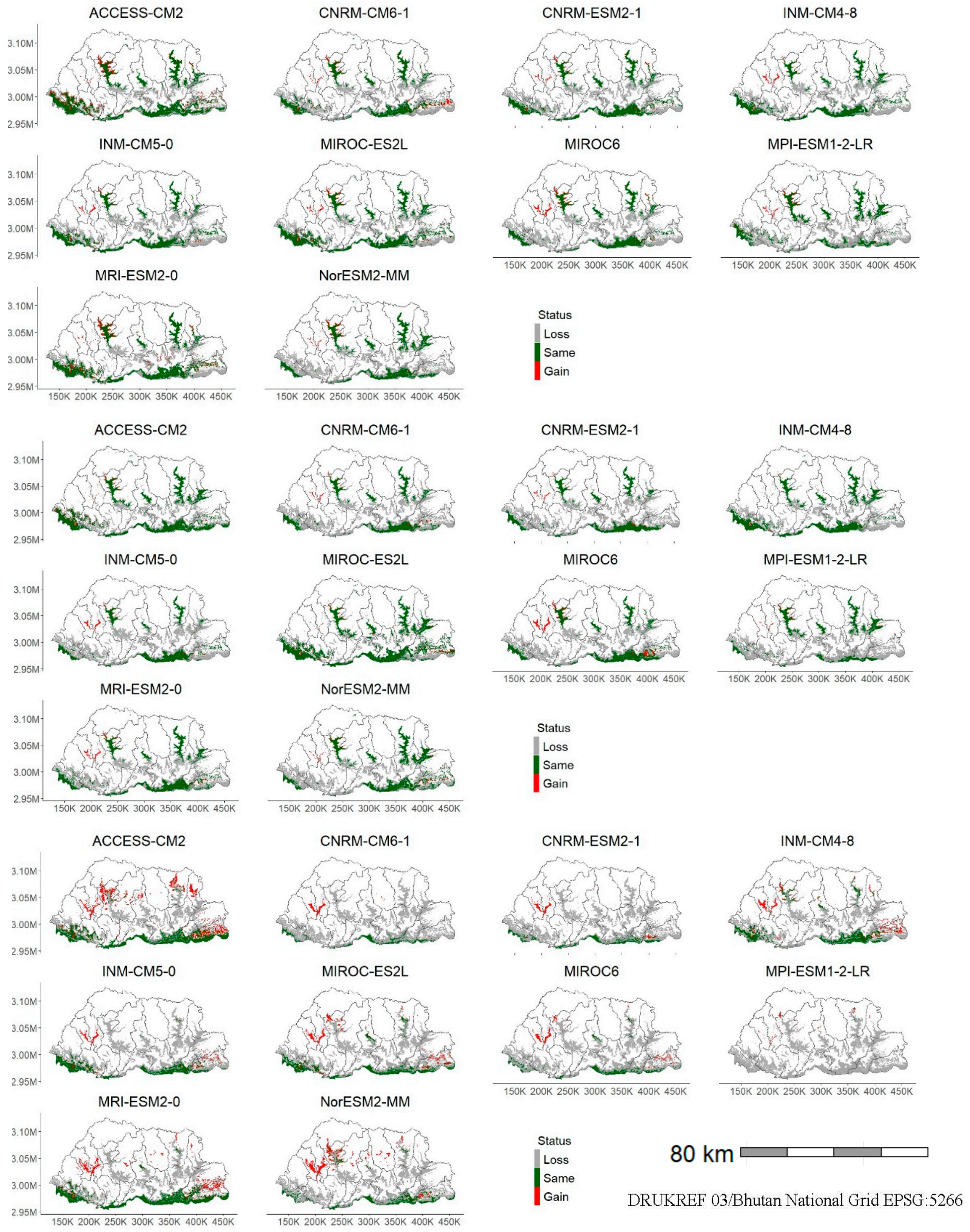

Note: "K" and "M" in the x and y-axis denote a thousand (x1000) and a million (x1000,000), respectively.

Figure S4. Maps of changes in predicted suitability of parthenium weed in Bhutan in the future. From the top: row 1 – 3, SSP126 in 2021-2050, 2051-2080, 2071-2100 periods; row 4 – 6, SSP245 in 2021-2050, 2051-2080, 2071-2100 periods; row 7 – 9, SSP370 in 2021-2050, 2051-2080, 2071-2100 periods; and row 10 – 12, SSP585 in 2021-2050, 2051-2080, 2071-2100 periods. Gray regions represent areas where the weed's distribution is predicted to lose, while green and red regions highlight areas of no change and gain in distribution, respectively. Notably, new areas of high suitability were predicted along highways in Paro and Thimphu.

Table S7. Predicted suitability of agricultural land to parthenium weed in the future under the four scenarios, SSP126, SSP245, SSP370, and SSP585, and the three periods, 2021-2050, 2051-2080, and 2071-2100 in Bhutan. Figures in the brackets indicate percentages of the predicted agricultural land suitability from the total suitability predicted by each general circulation model.

Model	Predicted suitability (km2) of agricultural land			
	SSP126	SSP245	SSP370	SSP585
	2021-2050			
ACCESS-CM2	1252.31 (31.04)	1186.94 (30.33)	949.5 (30.66)	1087.06 (31.01)
CNRM-ESM2-1	820.81 (34.36)	1277.88 (30.92)	885.06 (30.9)	698.06 (32.67)
CNRM-CM6-1	814.06 (33.9)	822.12 (31.08)	676.5 (30.45)	474.81 (30.08)
INM-CM4-8	1094.12 (32.96)	1029.5 (33.91)	1057.44 (31.33)	1134.31 (33.82)
INM-CM5-0	720.69 (32.87)	763.44 (33.43)	597.75 (30.05)	847.0 (33.06)
MIROC6	1444.19 (30.27)	1135.75 (31.98)	818.88 (34.63)	1141.94 (32.01)
MIROC-ES2L	918.38 (31.97)	676.81 (32.54)	532.94 (30.64)	862.38 (35.51)
MPI-ESM1-2-LR	727.06 (35.18)	860.75 (31.42)	799.44 (32.31)	762.56 (33.29)
MRI-ESM2-0	1007.31 (33.88)	1068.88 (32.39)	1292.44 (33.74)	886.75 (35.18)
NorESM2-MM	1007.88 (32.56)	1514.5 (30.96)	763.19 (30.7)	1607.56 (29.96)
	2051-2080			
ACCESS-CM2	1419.81 (28.29)	816.19 (28.65)	690.19 (28.01)	993.5 (27.14)
CNRM-ESM2-1	966.12 (32.37)	492.25 (33.84)	784.0 (31.14)	441.56 (31.32)
CNRM-CM6-1	976.69 (30.32)	564.38 (32.16)	276.19 (37.25)	218.44 (33.51)
INM-CM4-8	1003.31 (34.1)	1105.0 (33.02)	995.81 (32.77)	936.06 (34.14)
INM-CM5-0	1062.38 (33.14)	1298.5 (31.37)	694.88 (32.53)	742.88 (28.71)
MIROC6	1216.81 (31.21)	987.94 (32.42)	530.44 (37.13)	1013.5 (30.27)
MIROC-ES2L	872.94 (33.05)	536.38 (30.19)	450.06 (30.69)	229.56 (29.6)
MPI-ESM1-2-LR	953.19 (36.07)	474.94 (40.24)	325.25 (47.0)	209.12 (37.57)
MRI-ESM2-0	1287.06 (30.47)	968.25 (31.99)	542.06 (28.92)	791.94 (27.49)
NorESM2-MM	922.19 (32.53)	773.69 (33.0)	1381.06 (28.38)	398.88 (35.49)
	2071-2100			
ACCESS-CM2	1306.0 (28.22)	682.81 (25.1)	629.75 (24.87)	1006.94 (23.14)
CNRM-ESM2-1	655.69 (30.76)	301.62 (38.86)	339.88 (30.56)	331.06 (36.76)
CNRM-CM6-1	797.25 (30.99)	462.62 (31.23)	179.44 (32.25)	335.81 (31.66)
INM-CM4-8	1062.38 (33.14)	1298.5 (31.37)	694.88 (32.53)	742.88 (28.71)
INM-CM5-0	720.69 (32.87)	763.44 (33.43)	597.75 (30.05)	847.0 (33.06)
MIROC6	1603.12 (29.64)	862.5 (33.19)	335.25 (33.57)	645.12 (27.23)
MIROC-ES2L	1325.44 (31.42)	849.5 (31.74)	156.75 (33.55)	306.81 (25.42)
MPI-ESM1-2-LR	795.31 (39.13)	314.0 (44.5)	162.75 (43.63)	28.06 (19.62)
MRI-ESM2-0	1335.44 (29.85)	1163.81 (31.65)	678.31 (26.1)	951.06 (25.84)
NorESM2-MM	1118.94 (30.84)	697.81 (32.24)	1047.38 (27.8)	646.69 (29.48)

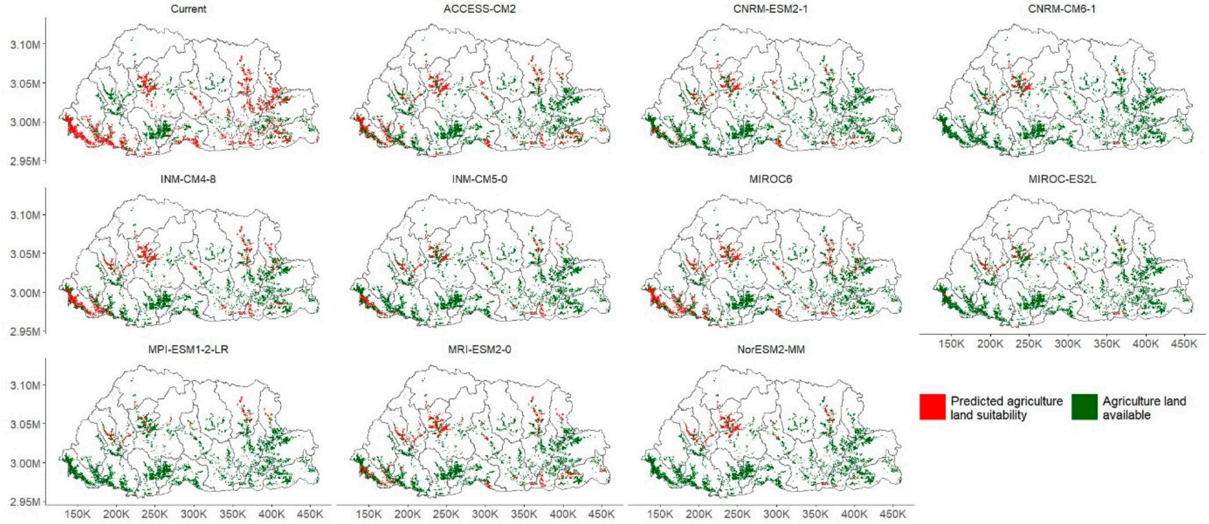
Current
ACCESS-CM2
CNRM-ESM2-1
CNRM-CM6-1
INM-CM4-8
INM-CM5-0
MIROC6
MIROC-ES2L
MPI-ESM1-2-LR
MRI-ESM2-0
NorESM2-MM
3.10M
3.05M
3.00M
2.95M
150K 200K 250K 300K 350K 400K 450K
Predicted agriculture land suitability
Agriculture land available

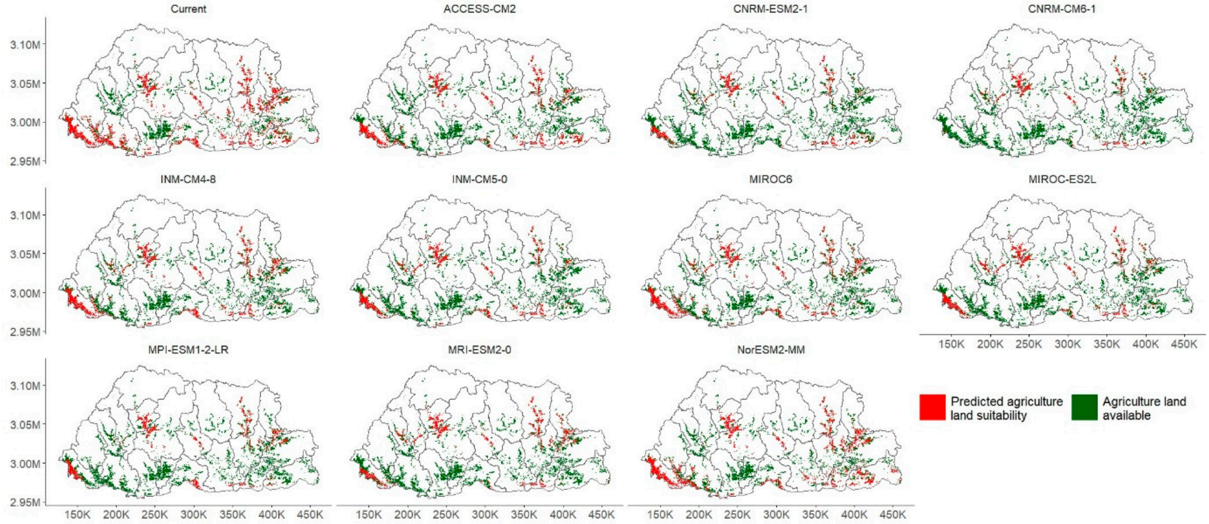
Current
ACCESS-CM2
CNRM-ESM2-1
CNRM-CM6-1
INM-CM4-8
INM-CM5-0
MIROC6
MIROC-ES2L
MPI-ESM1-2-LR
MRI-ESM2-0
NorESM2-MM
3.10M
3.05M
3.00M
2.95M
150K 200K 250K 300K 350K 400K 450K
Predicted agriculture land suitability
Agriculture land available

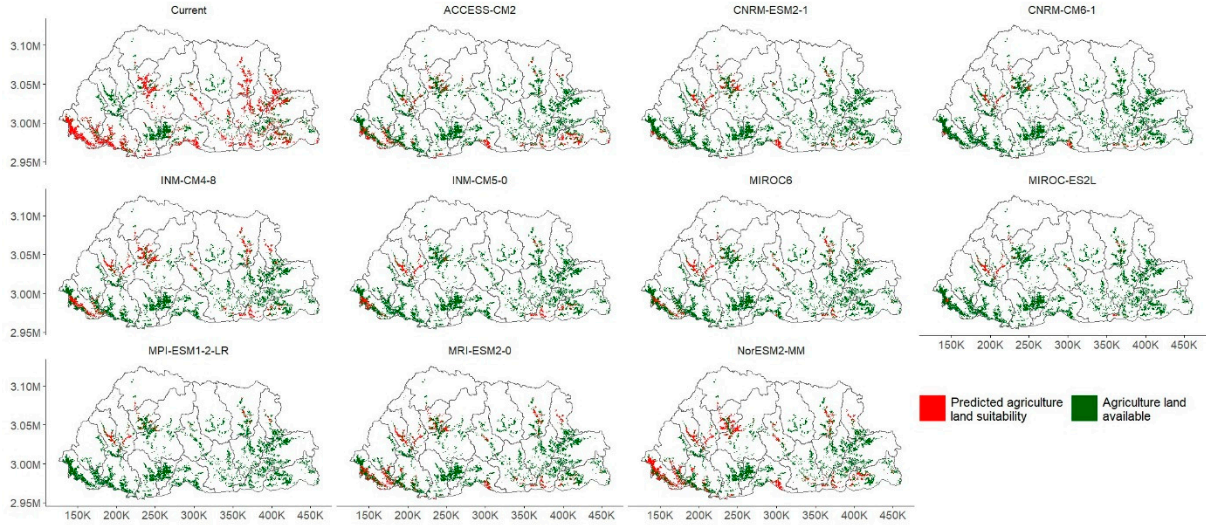
Current
ACCESS-CM2
CNRM-ESM2-1
CNRM-CM6-1
INM-CM4-8
INM-CM5-0
MIROC6
MIROC-ES2L
MPI-ESM1-2-LR
MRI-ESM2-0
NorESM2-MM
3.10M
3.05M
3.00M
2.95M
150K 200K 250K 300K 350K 400K 450K
Predicted agriculture land suitability
Agriculture land available

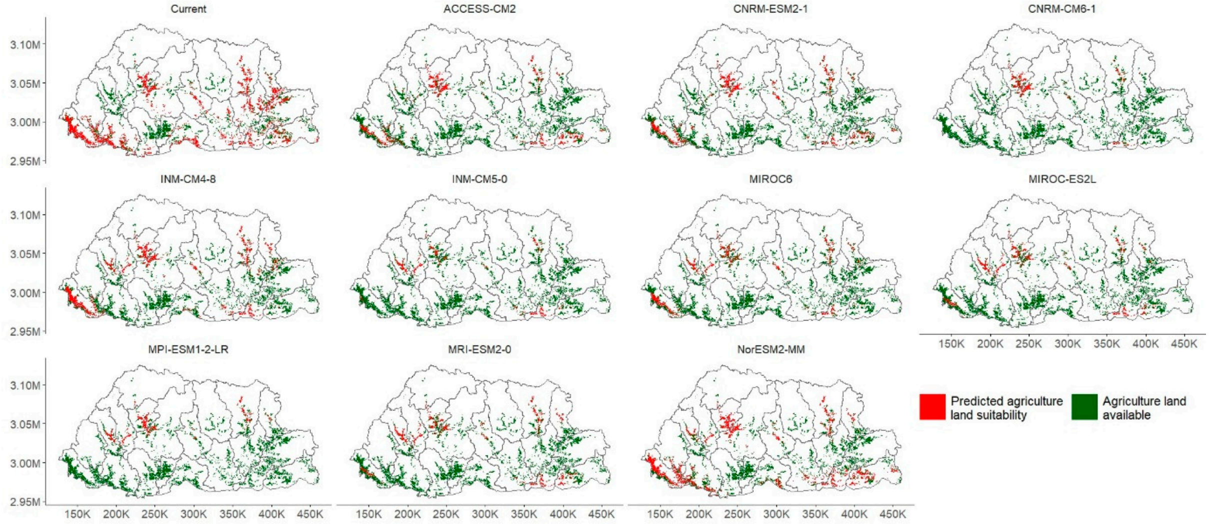
Current
ACCESS-CM2
CNRM-ESM2-1
CNRM-CM6-1
INM-CM4-8
INM-CM5-0
MIROC6
MIROC-ES2L
MPI-ESM1-2-LR
MRI-ESM2-0
NorESM2-MM
3.10M
3.05M
3.00M
2.95M
150K 200K 250K 300K 350K 400K 450K
Predicted agriculture land suitability
Agriculture land available

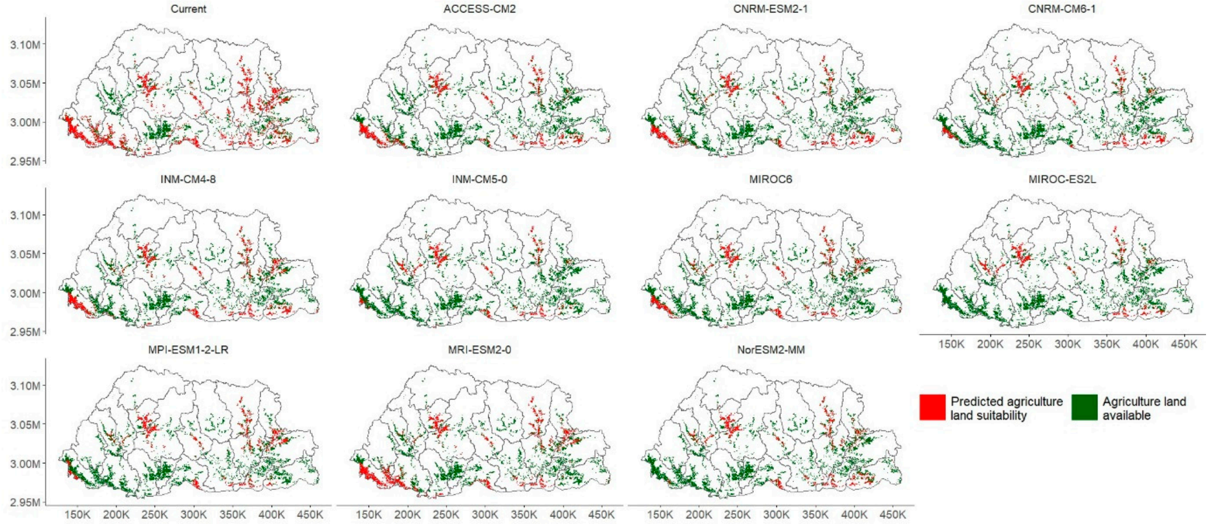
Current
ACCESS-CM2
CNRM-ESM2-1
CNRM-CM6-1
INM-CM4-8
INM-CM5-0
MIROC6
MIROC-ES2L
MPI-ESM1-2-LR
MRI-ESM2-0
NorESM2-MM
3.10M
3.05M
3.00M
2.95M
150K 200K 250K 300K 350K 400K 450K
Predicted agriculture land suitability
Agriculture land available

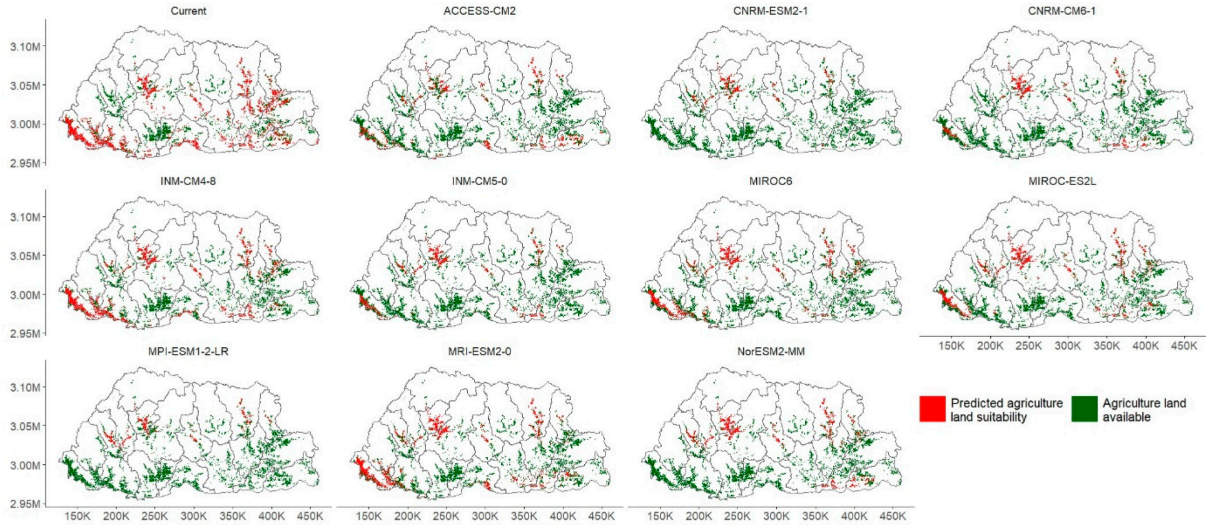
Current
ACCESS-CM2
CNRM-ESM2-1
CNRM-CM6-1
INM-CM4-8
INM-CM5-0
MIROC6
MIROC-ES2L
MPI-ESM1-2-LR
MRI-ESM2-0
NorESM2-MM
3.10M
3.05M
3.00M
2.95M
150K 200K 250K 300K 350K 400K 450K
Predicted agriculture land suitability
Agriculture land available

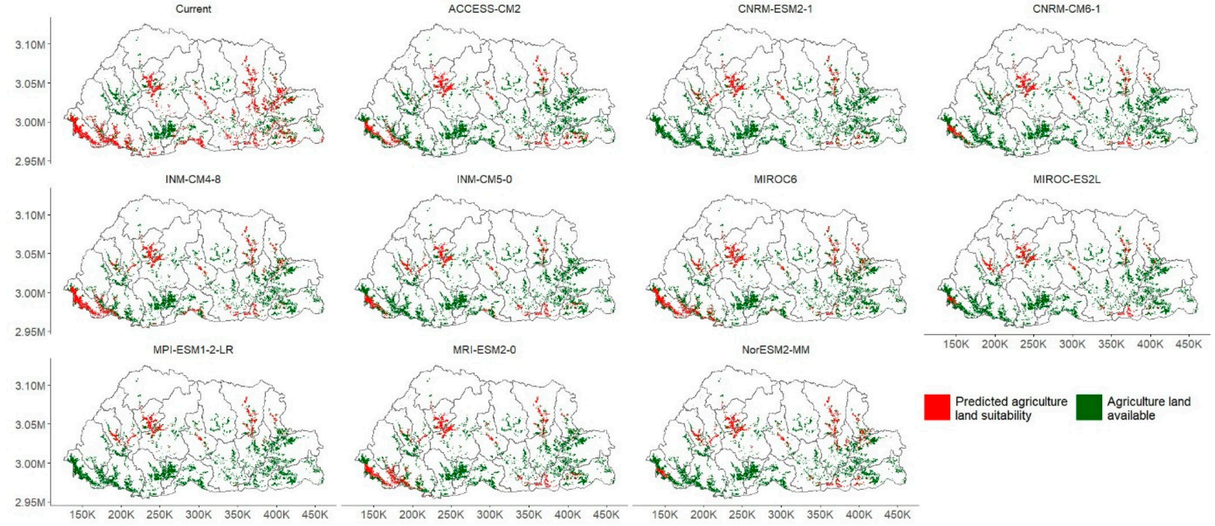
Current
ACCESS-CM2
CNRM-ESM2-1
CNRM-CM6-1
INM-CM4-8
INM-CM5-0
MIROC6
MIROC-ES2L
MPI-ESM1-2-LR
MRI-ESM2-0
NorESM2-MM
3.10M
3.05M
3.00M
2.95M
150K 200K 250K 300K 350K 400K 450K
Predicted agriculture land suitability
Agriculture land available

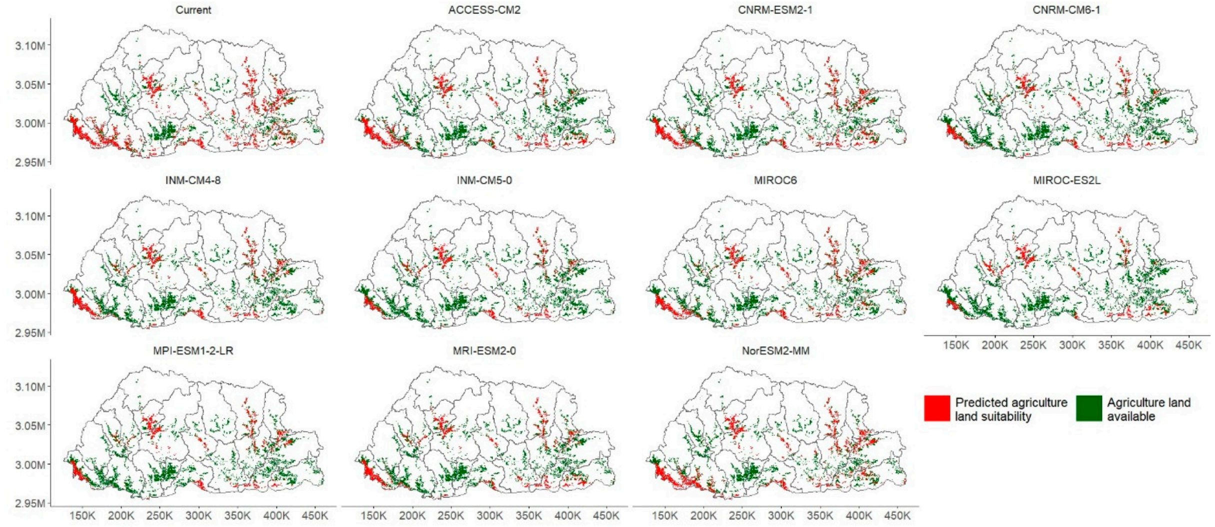
Current
ACCESS-CM2
CNRM-ESM2-1
CNRM-CM6-1
INM-CM4-8
INM-CM5-0
MIROC6
MIROC-ES2L
MPI-ESM1-2-LR
MRI-ESM2-0
NorESM2-MM
3.10M
3.05M
3.00M
2.95M
150K 200K 250K 300K 350K 400K 450K
Predicted agriculture land suitability
Agriculture land available

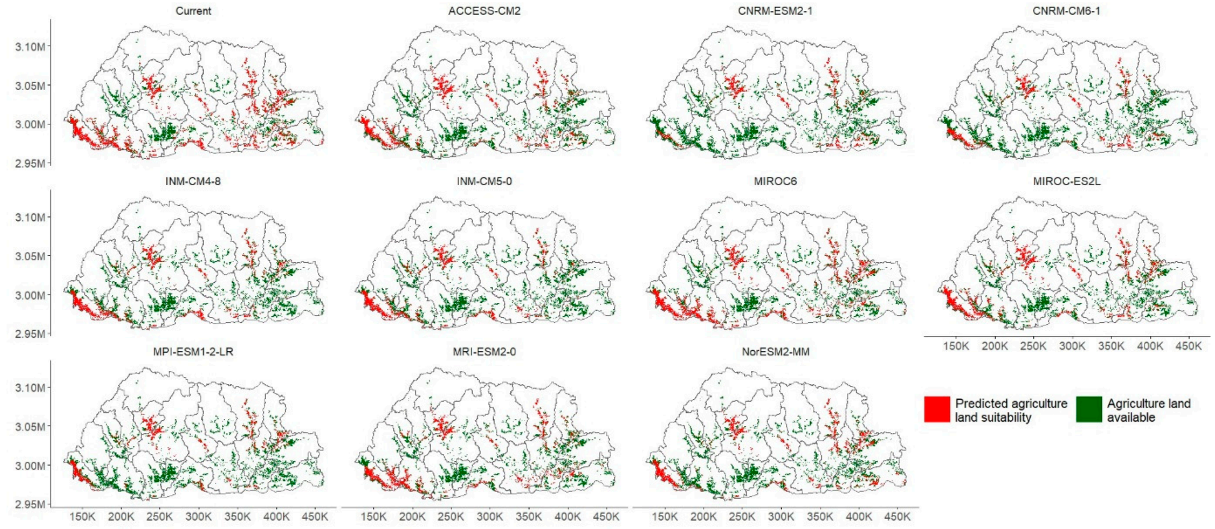
Current
ACCESS-CM2
CNRM-ESM2-1
CNRM-CM6-1
INM-CM4-8
INM-CM5-0
MIROC6
MIROC-ES2L
MPI-ESM1-2-LR
MRI-ESM2-0
NorESM2-MM
3.10M
3.05M
3.00M
2.95M
150K 200K 250K 300K 350K 400K 450K
Predicted agriculture land suitability
Agriculture land available

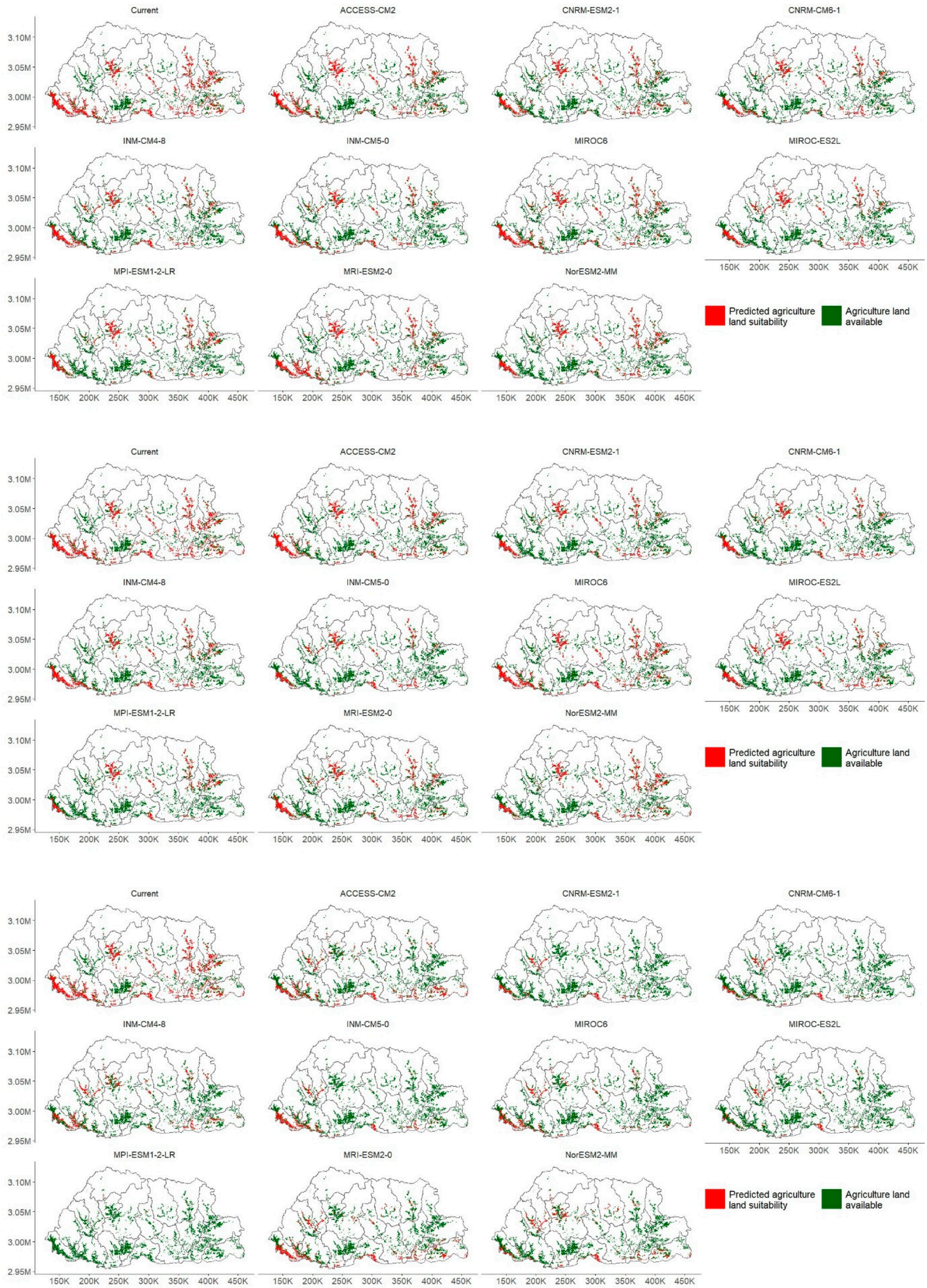

Figure S5. Maps of predicted agricultural land suitability (red) to parthenium weed against the total agricultural land available (green) in the country. From the top: row 1 – 3, SSP126 in 2021-

2050, 2051-2080, 2071-2100 periods; row 4 – 6, SSP245 in 2021-2050, 2051-2080, 2071-2100 periods; row 7 – 9, SSP370 in 2021-2050, 2051-2080, 2071-2100 periods; and row 10 – 12, SSP585 in 2021-2050, 2051-2080, 2071-2100 periods.

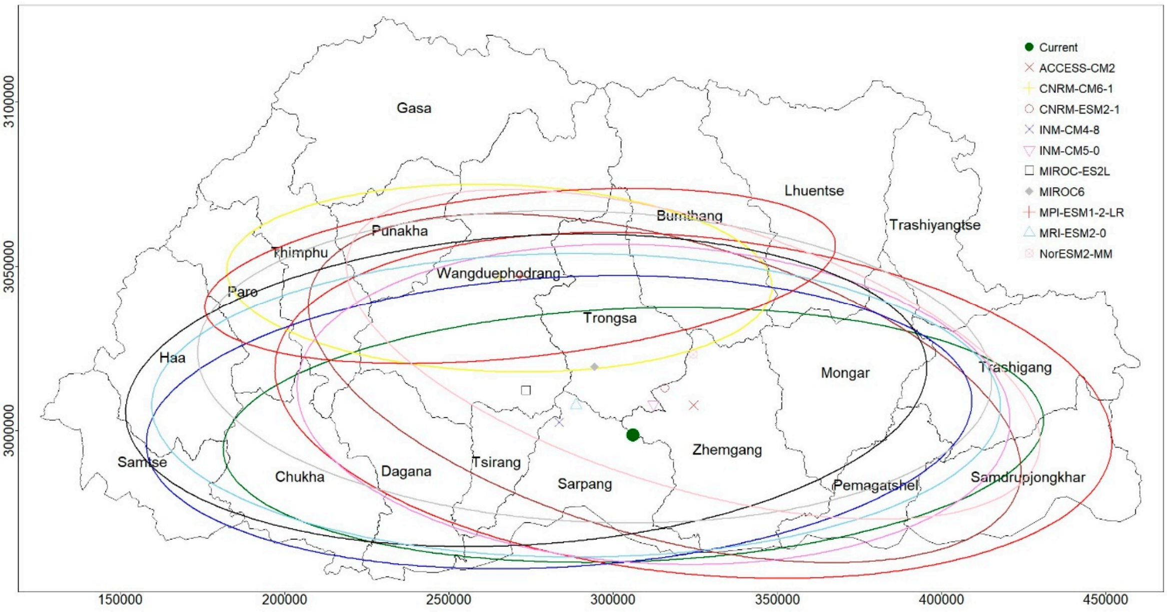

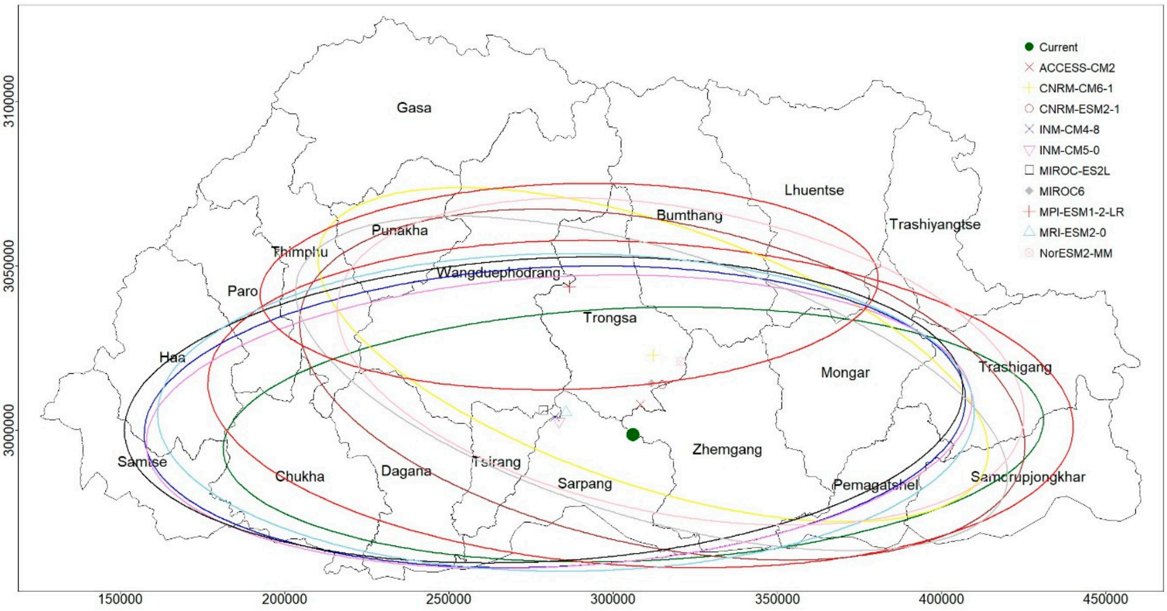

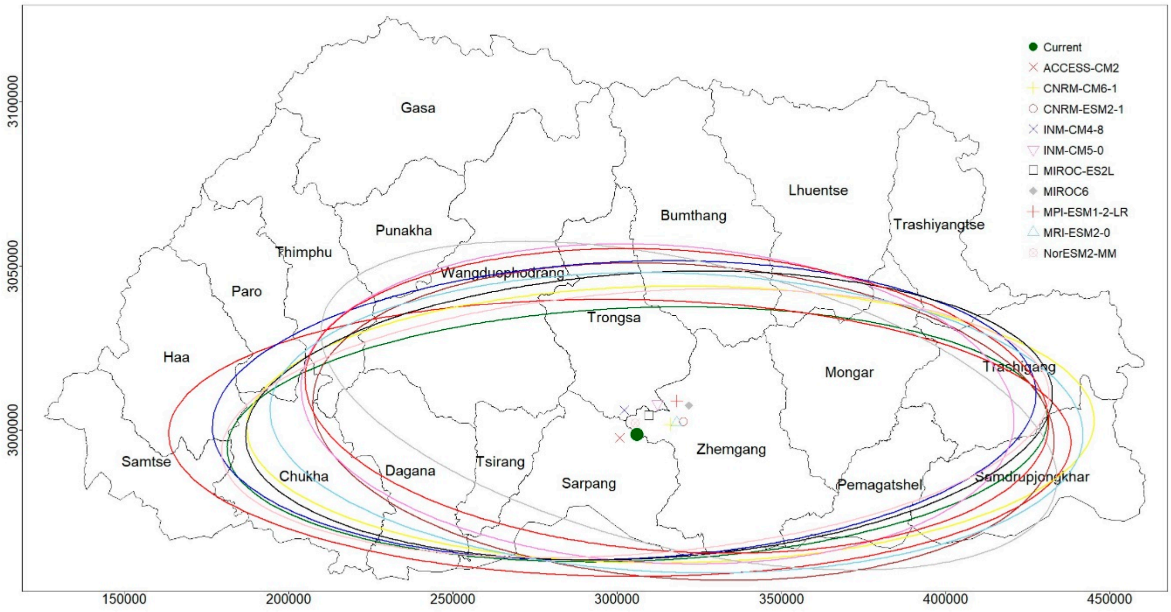
Current
ACCESS-CM2
CNRM-CM6-1
CNRM-ESM2-1
INM-CM4-8
INM-CM5-0
MIROC-ES2L
MIROC6
MPI-ESM1-2-LR
MRI-ESM2-0
NorESM2-MM
Gasa
Lhuentse
Bumthang
Trashiyangtse
Punakha
Thimphu
Paro
Wangduephodrang
Trongsa
Haa
Mongar
Trashigang
Samtse
Chukha
Dagana
Tsirang
Sarpang
Zhemgang
Pemagatshel
Samdrupjongkhar
3100000
3050000
3000000
150000
200000
250000
300000
350000
400000
450000

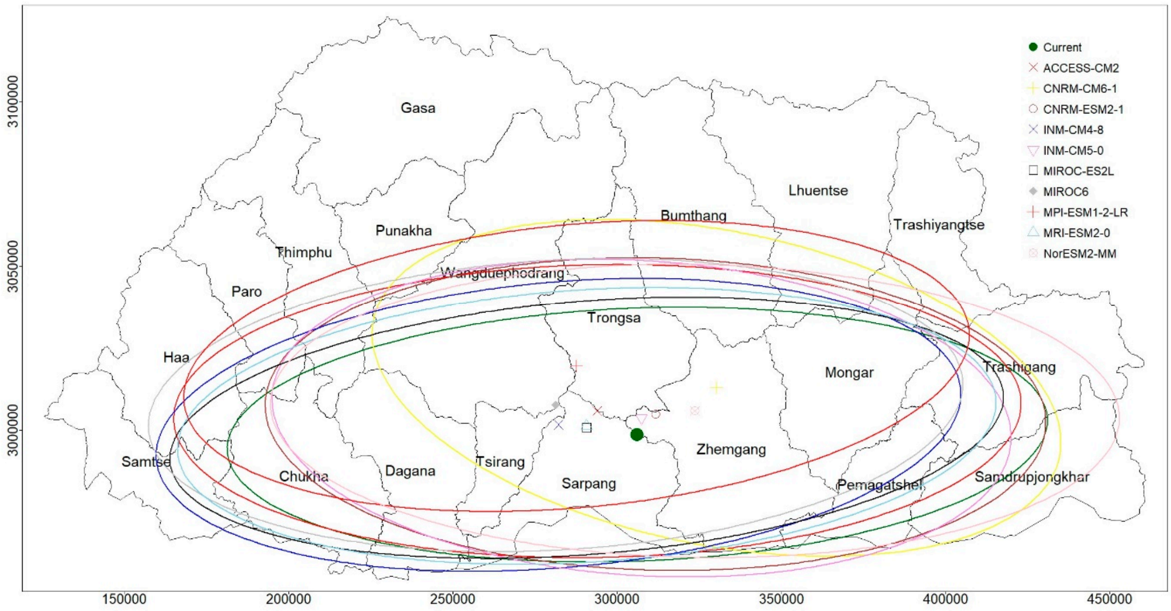
Current
ACCESS-CM2
CNRM-CM6-1
CNRM-ESM2-1
INM-CM4-8
INM-CM5-0
MIROC-ES2L
MIROC6
MPI-ESM1-2-LR
MRI-ESM2-0
NorESM2-MM
Gasa
Lhuentse
Bumthang
Trashiyangtse
Punakha
Thimphu
Paro
Wangduephodrang
Trongsa
Haa
Mongar
Trashigang
Samtse
Chukha
Dagana
Tsirang
Sarpang
Zhemgang
Pemagatshel
Samdrupjongkhar
3100000
3050000
3000000
150000
200000
250000
300000
350000
400000
450000

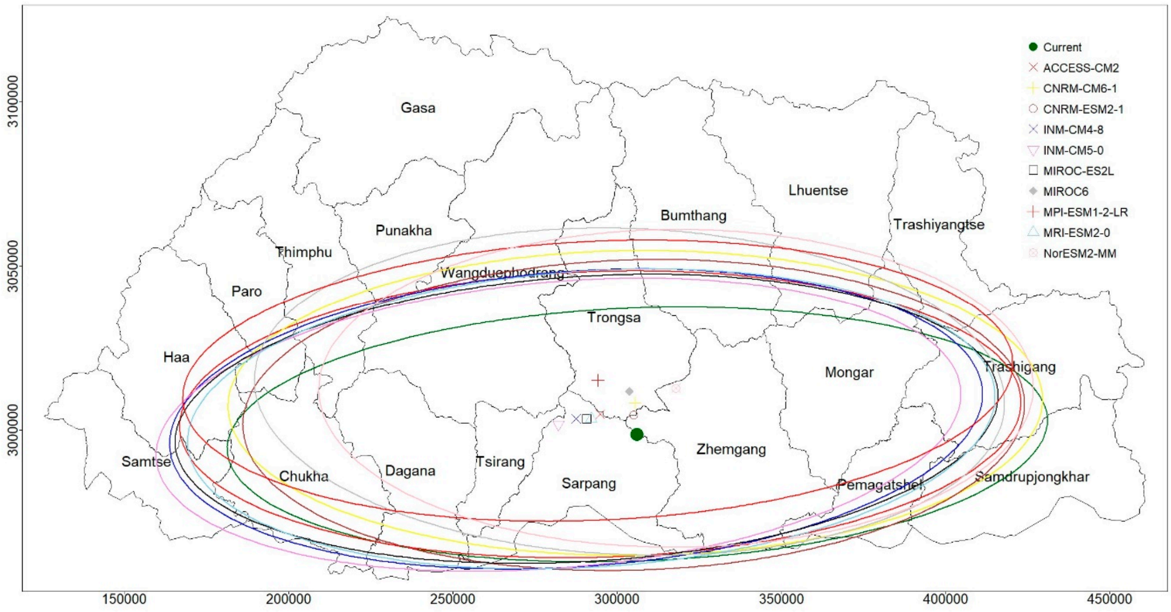
Current
ACCESS-CM2
CNRM-CM6-1
CNRM-ESM2-1
INM-CM4-8
INM-CM5-0
MIROC-ES2L
MIROC6
MPI-ESM1-2-LR
MRI-ESM2-0
NorESM2-MM
Gasa
Lhuentse
Bumthang
Trashiyangtse
Punakha
Thimphu
Wangduephodrang
Paro
Trongsa
Haa
Mongar
Trashigang
Samtse
Zhemgang
Chukha
Dagana
Tsirang
Sarpang
Pemagatshel
Samdrupjongkhar
3100000
3050000
3000000
150000
200000
250000
300000
350000
400000
450000

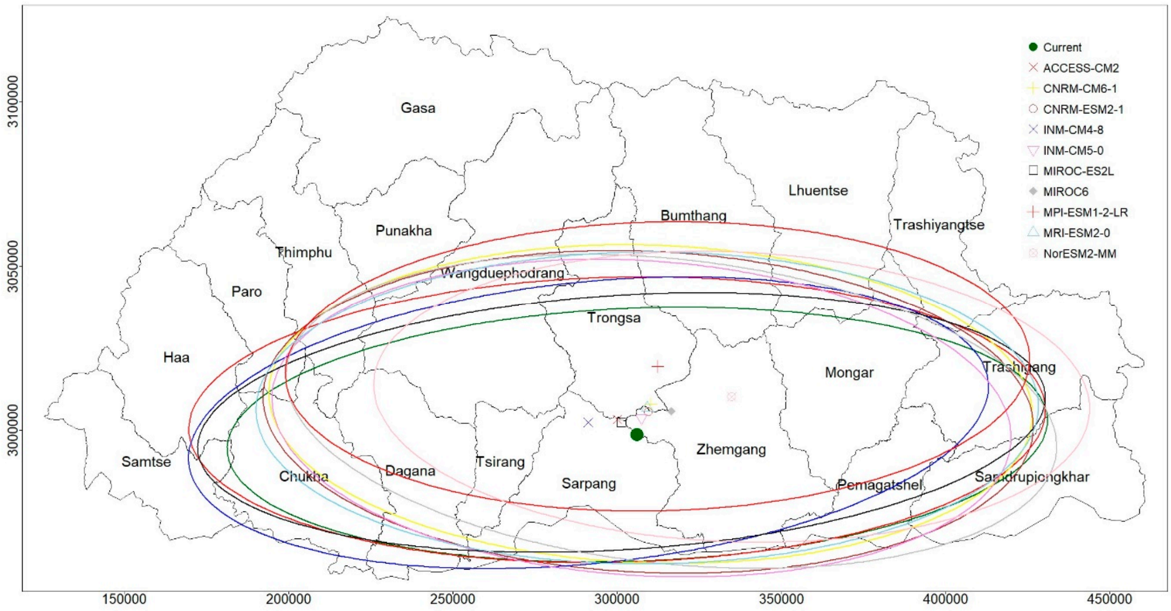
Current
ACCESS-CM2
CNRM-CM6-1
CNRM-ESM2-1
INM-CM4-8
INM-CM5-0
MIROC-ES2L
MIROC6
MPI-ESM1-2-LR
MRI-ESM2-0
NorESM2-MM
Gasa
Lhuentse
Bumthang
Trashiyangtse
Punakha
Thimphu
Wangduephodrang
Paro
Trongsa
Haa
Mongar
Trashigang
Samtse
Zhemgang
Chukha
Dagana
Tsirang
Sarpang
Pemagatshel
Samdrupjongkhar
3100000
3050000
3000000
150000
200000
250000
300000
350000
400000
450000

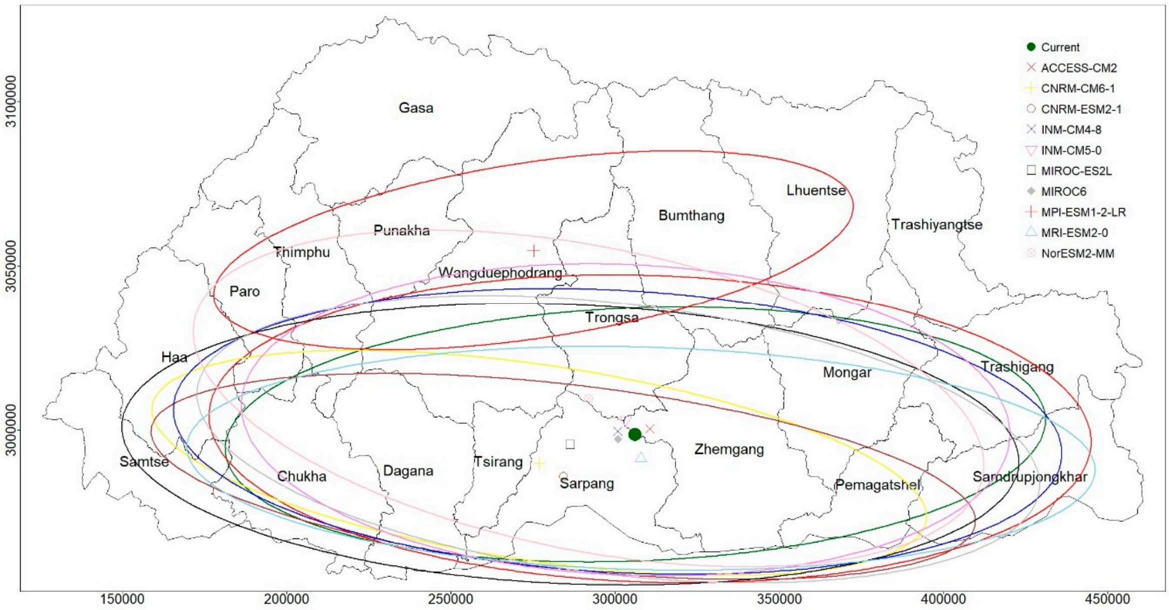
Current
ACCESS-CM2
CNRM-CM6-1
CNRM-ESM2-1
INM-CM4-8
INM-CM5-0
MIROC-ES2L
MIROC6
MPI-ESM1-2-LR
MRI-ESM2-0
NorESM2-MM
Gasa
Lhuentse
Bumthang
Trashiyangtse
Punakha
Thimphu
Wangduephodrang
Paro
Trongsa
Haa
Mongar
Trashigang
Samtse
Chukha
Dagana
Tsirang
Sarpang
Zhemgang
Pemagatshel
Samdrupjongkhar
3100000
3050000
3000000
150000
200000
250000
300000
350000
400000
450000

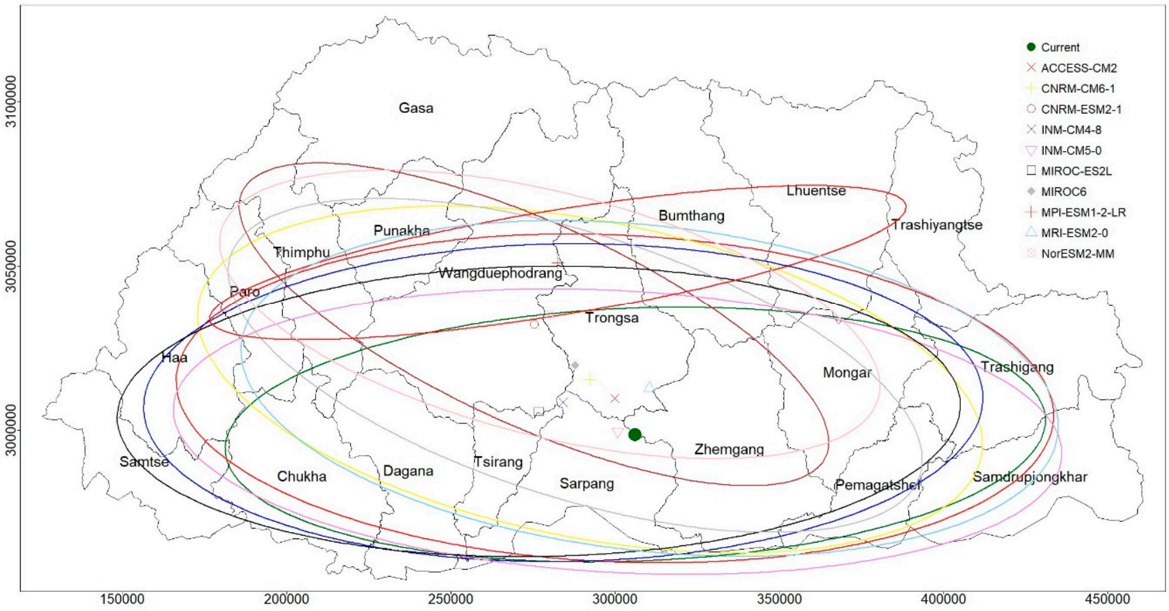
Current
ACCESS-CM2
CNRM-CM6-1
CNRM-ESM2-1
INM-CM4-8
INM-CM5-0
MIROC-ES2L
MIROC6
MPI-ESM1-2-LR
MRI-ESM2-0
NorESM2-MM
Gasa
Lhuentse
Bumthang
Trashiyangtse
Punakha
Thimphu
Wangduephodrang
Paro
Trongsa
Haa
Mongar
Trashigang
Samtse
Chukha
Dagana
Tsirang
Sarpang
Zhemgang
Pemagatshel
Samdrupjongkhar
3100000
3050000
3000000
150000
200000
250000
300000
350000
400000
450000

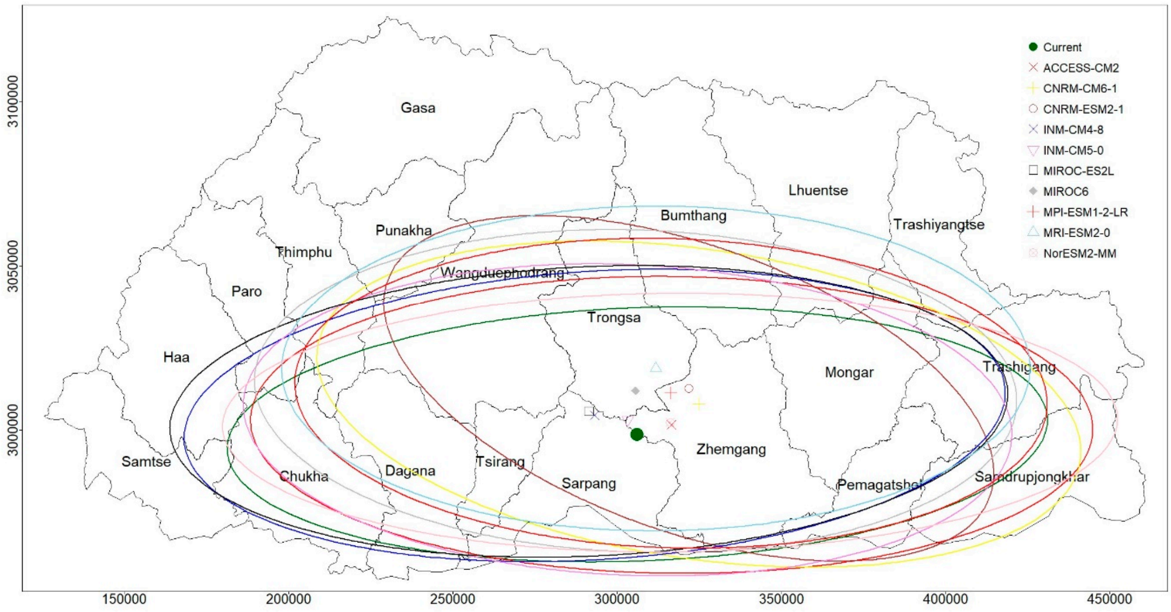
Current
ACCESS-CM2
CNRM-CM6-1
CNRM-ESM2-1
INM-CM4-8
INM-CM5-0
MIROC-ES2L
MIROC6
MPI-ESM1-2-LR
MRI-ESM2-0
NorESM2-MM
Gasa
Lhuentse
Bumthang
Trashiyangtse
Punakha
Thimphu
Wangduephodrang
Paro
Trongsa
Haa
Mongar
Trashigang
Samtse
Zhemgang
Chukha
Dagana
Tsirang
Sarpang
Pemagatshel
Samdrupjongkhar
3100000
3050000
3000000
150000
200000
250000
300000
350000
400000
450000

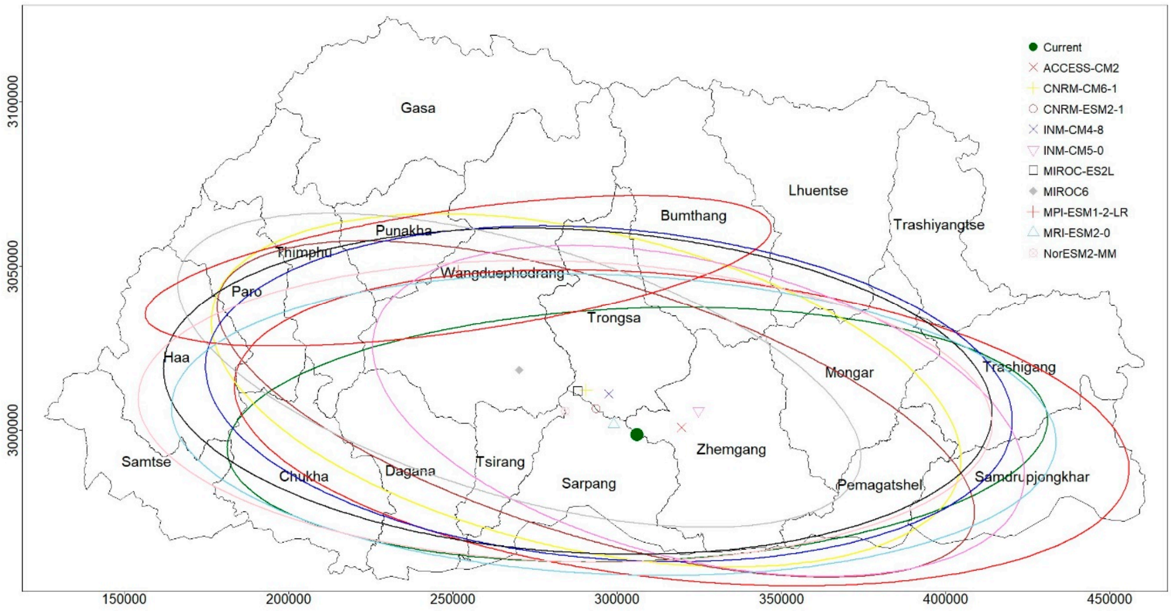
Current
ACCESS-CM2
CNRM-CM6-1
CNRM-ESM2-1
INM-CM4-8
INM-CM5-0
MIROC-ES2L
MIROC6
MPI-ESM1-2-LR
MRI-ESM2-0
NorESM2-MM
Gasa
Lhuentse
Bumthang
Trashiyangtse
Punakha
Thimphu
Wangduephodrang
Paro
Trongsa
Haa
Mongar
Trashigang
Samtse
Zhemgang
Chukha
Dagana
Tsirang
Sarpang
Pemagatshel
Samdrupjongkhar
3100000
3050000
3000000
150000
200000
250000
300000
350000
400000
450000

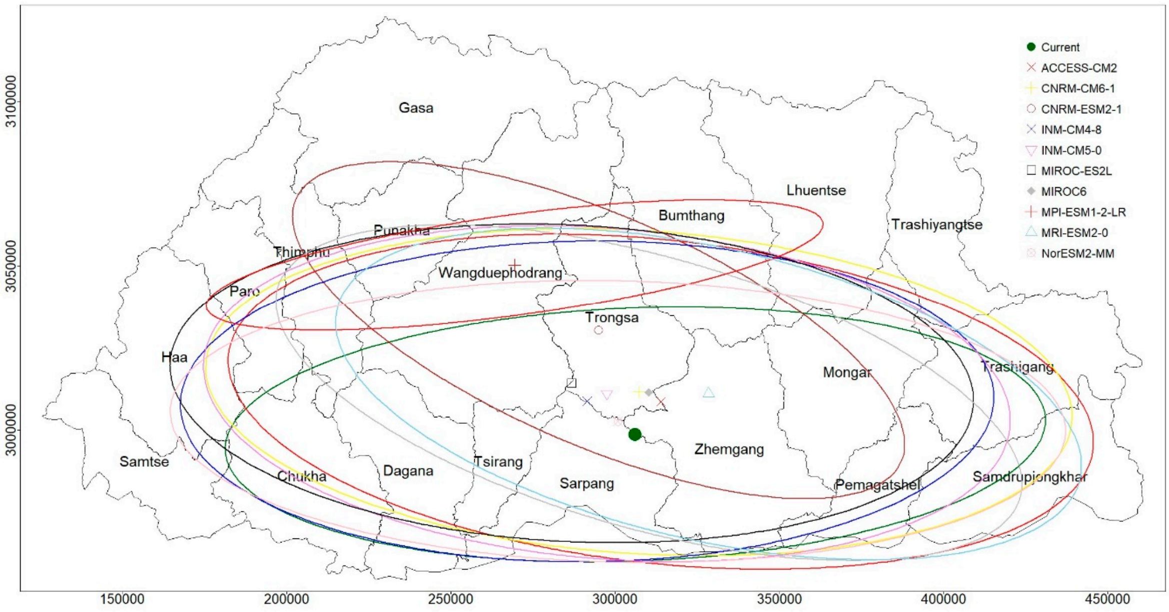

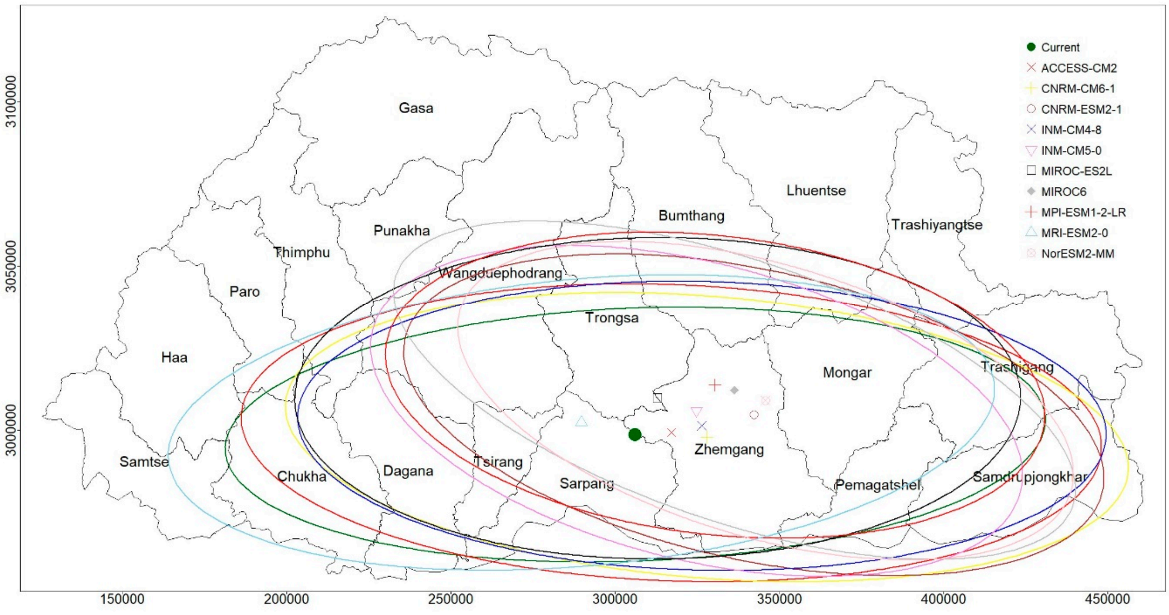

Figure S6. The standard deviational ellipse (SDE) and the mean location of parthenium weed distribution under the current and future climate. From the top: row 1-3, SSP126 in 2021-2050, 2051-2080, 2071-2100 periods; row 4-6, SSP245 in 2021-2050, 2051-2080, 2071-2100 periods; row 7-9, SSP370 in 2021-2050, 2051-2080, 2071-2100 periods; and row 10-12, SSP585 in 2021-2050, 2051-2080, 2071-2100 periods. Different colours represent the ellipses for various general circulation models (GCMs), with each model's mean location marked by distinct symbols.

Table S8. Shift in the mean location (km) of parthenium weed distribution in the future under four scenarios, SSP126, SSP245, SSP370, SSP585 and three periods, 2021-2050, 2051-2080, and 2071-2100.

Model	Shift in mean location (km)			
	SSP126	SSP245	SSP370	SSP585
	2021-2050			
ACCESS-CM2	8.02	8.18	9.80	8.91
CNRM-CM6-1	7.29	8.57	20.73	18.60
CNRM-ESM2-1	4.63	12.60	34.97	18.58
INM-CM4-8	16.70	7.41	18.83	15.05
INM-CM5-0	2.30	8.27	17.80	4.88
MIROC-ES2L	6.56	3.66	10.22	17.09
MIROC6	9.89	15.57	31.04	11.32
MPI-ESM1-2-LR	19.73	13.15	26.36	13.54
MRI-ESM2-0	5.77	10.46	18.21	18.92
NorESM2-MM	28.90	2.30	39.23	8.47
	2051-2080			
ACCESS-CM2	13.48	6.80	9.82	11.77
CNRM-CM6-1	7.53	23.41	10.76	21.56
CNRM-ESM2-1	4.25	15.05	33.61	46.51
INM-CM4-8	20.57	25.88	18.15	24.87
INM-CM5-0	25.87	24.48	14.57	7.38
MIROC-ES2L	17.33	29.62	25.17	31.63
MIROC6	11.65	14.17	10.89	28.02
MPI-ESM1-2-LR	20.01	50.05	65.13	58.97
MRI-ESM2-0	15.87	22.70	23.23	12.31
NorESM2-MM	15.78	24.42	7.04	45.75
	2071-2100			
ACCESS-CM2	14.63	18.07	12.05	3.18
CNRM-CM6-1	25.80	64.43	20.76	33.57
CNRM-ESM2-1	5.35	14.58	15.29	29.21
INM-CM4-8	25.87	24.48	14.57	7.38
INM-CM5-0	2.30	8.27	17.80	4.88
MIROC-ES2L	17.21	36.43	22.82	22.52
MIROC6	27.54	23.49	41.95	8.32
MPI-ESM1-2-LR	28.05	60.95	75.91	65.97
MRI-ESM2-0	17.23	20.32	8.88	11.03
NorESM2-MM	16.71	28.67	24.23	18.20
